# Structure of *S. aureus* HPPK and the Discovery of a New Substrate Site Inhibitor

**DOI:** 10.1371/journal.pone.0029444

**Published:** 2012-01-19

**Authors:** Sandeep Chhabra, Olan Dolezal, Brett M. Collins, Janet Newman, Jamie S. Simpson, Ian G. Macreadie, Ross Fernley, Thomas S. Peat, James D. Swarbrick

**Affiliations:** 1 Medicinal Chemistry and Drug Action, Monash Institute of Pharmaceutical Sciences, Monash University, Parkville, Australia; 2 CSIRO Division of Materials, Science and Engineering, Parkville, Australia; 3 Institute for Molecular Bioscience, The University of Queensland, Australia; 4 School of Applied Sciences, RMIT University, Bundoora, Australia; University of Queensland, Australia

## Abstract

The first structural and biophysical data on the folate biosynthesis pathway enzyme and drug target, 6-hydroxymethyl-7,8-dihydropterin pyrophosphokinase (SaHPPK), from the pathogen *Staphylococcus aureus* is presented. HPPK is the second essential enzyme in the pathway catalysing the pyrophosphoryl transfer from cofactor (ATP) to the substrate (6-hydroxymethyl-7,8-dihydropterin, HMDP). In-silico screening identified 8-mercaptoguanine which was shown to bind with an equilibrium dissociation constant, K_d_, of ∼13 µM as measured by isothermal titration calorimetry (ITC) and surface plasmon resonance (SPR). An IC_50_ of ∼41 µM was determined by means of a luminescent kinase assay. In contrast to the biological substrate, the inhibitor has no requirement for magnesium or the ATP cofactor for competitive binding to the substrate site. The 1.65 Å resolution crystal structure of the inhibited complex showed that it binds in the pterin site and shares many of the key intermolecular interactions of the substrate. Chemical shift and ^15^N heteronuclear NMR measurements reveal that the fast motion of the pterin-binding loop (L2) is partially dampened in the SaHPPK/HMDP/α,β-methylene adenosine 5′-triphosphate (AMPCPP) ternary complex, but the ATP loop (L3) remains mobile on the µs-ms timescale. In contrast, for the SaHPPK/8-mercaptoguanine/AMPCPP ternary complex, the loop L2 becomes rigid on the fast timescale and the L3 loop also becomes more ordered – an observation that correlates with the large entropic penalty associated with inhibitor binding as revealed by ITC. NMR data, including ^15^N-^1^H residual dipolar coupling measurements, indicate that the sulfur atom in the inhibitor is important for stabilizing and restricting important motions of the L2 and L3 catalytic loops in the inhibited ternary complex. This work describes a comprehensive analysis of a new HPPK inhibitor, and may provide a foundation for the development of novel antimicrobials targeting the folate biosynthetic pathway.

## Introduction

*Staphylococcus aureus* is a clinically important opportunistic pathogen and one of the major contributors to hospital- and community-acquired bacterial infections. Methicillin-resistant *S. aureus* strains (MRSA, commonly referred to as the “superbug”) cause up to 19,000 deaths annually in the US alone, and an estimated health care cost of $ 3–4 billion per annum [1]. MRSA strains are classified by genotypic and phenotypic characteristics, and are grouped into two major categories: those originating in hospitals (nosocomial, haMRSA, strains USA100 and USA200) and those in the community (caMRSA), of which the latter is almost entirely caused by the pandemic USA300 strain [2]. Infection with USA300 causes abscesses and life threatening systemic infections, such as bacteremia and necrotizing pneumonia. In contrast to haMRSA, caMRSA infections tend to occur in previously healthy younger patients without health care exposure [3]. Currently, caMRSA is more susceptible to a range of chemotherapies than the multi-drug resistant haMRSA [4]. Although resistant to tetracycline, erythromycin, clindamycin, linezolid, and in some cases vancomycin, caMRSA is largely susceptible to TMP-SMX (trimethoprim-sulfamethoxazole) combination therapy, which synergistically blocks the biosynthesis of folate derivatives by acting on dihydrofolatereductase (DHFR) and dihydropteroatesynthase (DHPS), respectively [5], [6]. TMP-SMX resistance has emerged in haMRSA owing to an ‘autolytic’ thyamidine salvage pathway effective when polymerized DNA is released from damaged tissues [6], [7], [8]. TMP-SMX resistance in caMRSA is attributed to mutations in the DHFR or DHPS genes, which in the former case results in a repositioning of the substrate in the active site [9], compromising TMP-based therapy.

Classically, targets for antimicrobials are found to be essential enzymes that are unique to the micro-organism (not present in the host), and new antimicrobial drugs have been developed from molecules identified in proof-of-concept studies [10]. The folate biosynthetic pathway fits the criterion of being an attractive source of potential target enzymes, and antimicrobials against key components of this pathway are used today to treat diseases such as malaria, pneumocystis pneumonia (PCP) and caMRSA infections. Folates are essential for the growth of all living cells. The reduced form of folate, tetrahydrofolate (THF), participates in several important one-carbon transfers, critical for the biosynthesis of thymidine, glycine and methionine, and is vital for DNA replication [11], [12].

**Figure 1:**
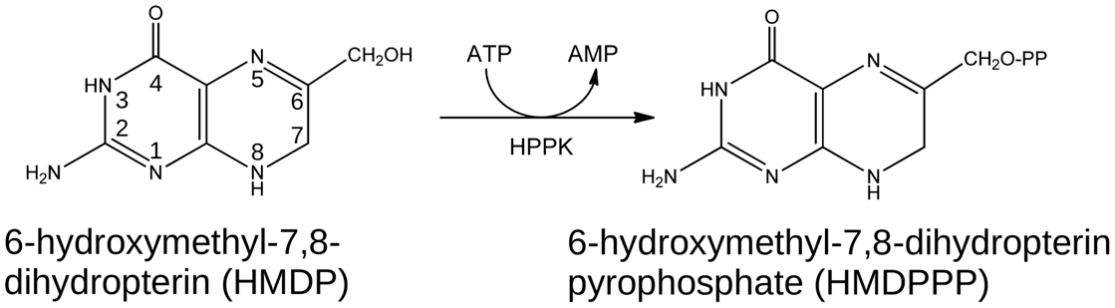
Pyrophosphoryl transfer catalysed by HPPK.

**Figure 2:**
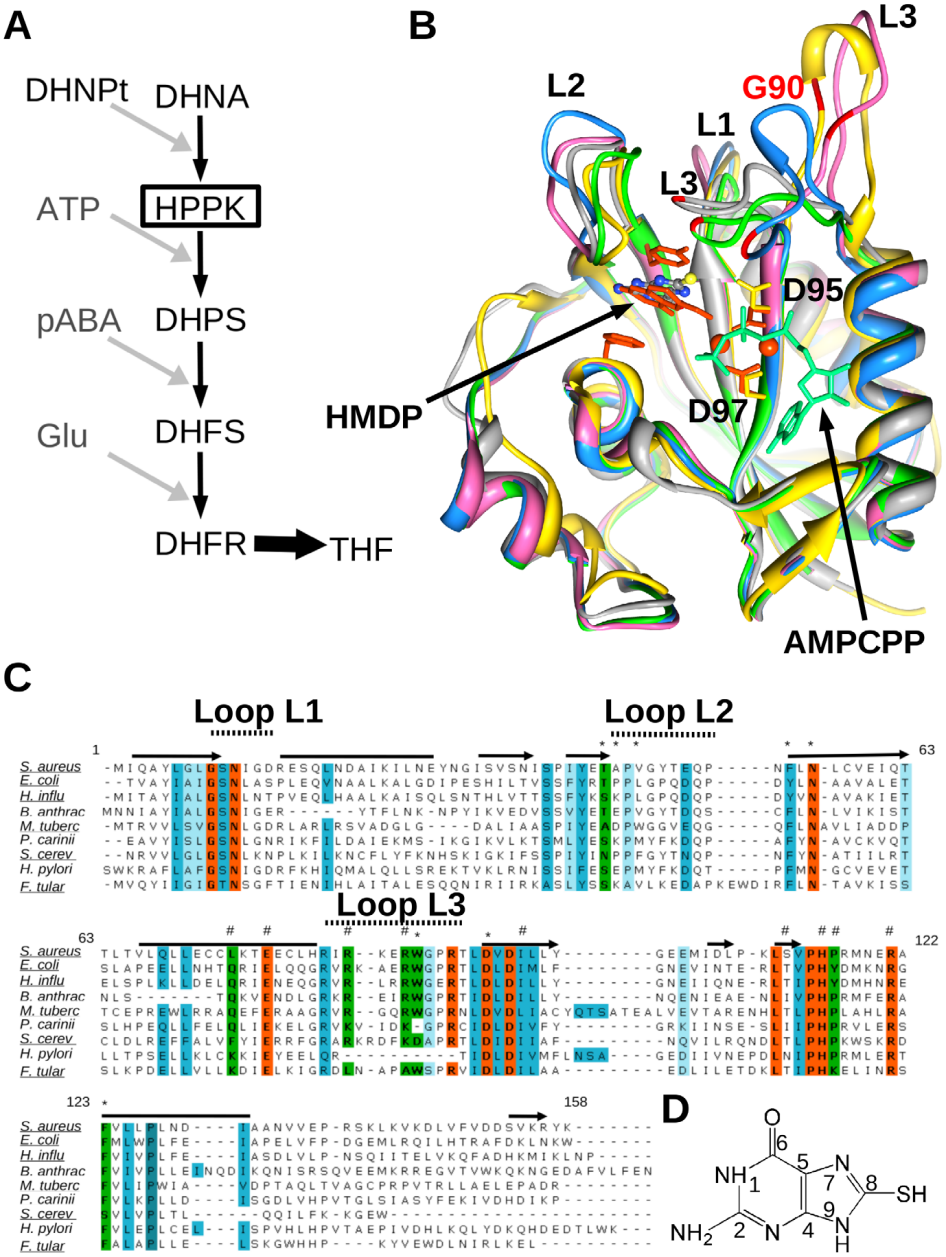
Folate pathway, HPPK structures and HPPK sequences. A, Schematic showing the folate pathway conversion of dihydroneopterin (DHNPt) into tetrahydrofolate (THF) via the five essential enzymes, DHNA, HPPK, DHPS, DHFS and DHFR. B, Superposition of selected HPPK structures in complex with HMDP (orange), 8-mercaptoguanine (elemental colouring) and AMPCPP (cyan), showing the prototypical HPPK fold, active site loops, Gly90 (red), the HPPK conserved ring stacking residues and magnesium ions (orange) bound by the conserved aspartate residues. SaHPPK (3QBC) from this study is shown (yellow). Three EcHPPK structures are shown in complex with AMPCPP and HMDP (1Q0N, green); apo (1HKA, blue); with HMDPP and AMP (1RAO, magenta) and HPPK from *H. Influenza* (1CBK, grey). C, Multiple sequence alignment of selected HPPKs. Absolutely conserved residues (orange), highly conserved residues (blue), and residues that are additionally important for HPPK function and may be targeted to develop selective inhibitors are shown (green). Residues involved in HMDP (*) and ATP recognition (#) and elements of secondary structure are displayed above the alignment. Underlined sequences have had their structure determined by x-ray crystallography. D, Structure of the SaHPPK inhibitor 8-mercaptoguanine.

6-Hydroxymethyl-7,8-dihydropterin pyrophosphokinase (HPPK, EC 2.7.6.3) catalyses pyrophosphoryl transfer from ATP (cofactor) to the substrate, 6-hydroxymethyl-7,8-dihydropterin (HMDP) (Fig. 1). HPPK is the upstream and adjacent enzyme to DHPS in the folate biosynthesis pathway (Fig. 2*A*). It is not the target of any existing drug and therefore represents an attractive resource for the rational design of novel antimicrobials and antifungals to act on current TMP-SMX-resistant isolates for the treatment of caMRSA infections. HPPK is a small (158 residues, ∼18 kDa), generally monomeric protein and has been studied using various biophysical techniques, including x-ray crystallography and NMR spectroscopy. A number of x-ray and NMR structures of HPPK have been determined in various ligand-bound states (Fig. 2*B*) and from a variety of organisms: *Escherichia coli*, *Haemophilusinfluenzae*, *Saccharomyces cerevisiae*, *Streptococcus pneumonia*, *Yersinia pestis* and *Francisella tularensis* [13], [14], [15], [16], [17], [18]. These data have provided atomic level information on the catalytic mechanism and protein dynamics of the reaction trajectory during catalysis [19]. Three loop regions, loops L1–3, play an important role in substrate recognition and are critical for assembling the active centre [20]. While loop L3 undergoes the largest and most dramatic conformational change during the catalytic cycle, all three loops help to seal the substrate and cofactor binding sites for the chemical transfer of a pyrophosphate from ATP to HMDP [21]. The substrate and cofactor interact with two magnesium ions and associate with a total of 26 residues in HPPK, 13 of which are conserved across all species [22]. *In vitro* kinetic studies have shown a preferred order of substrate binding. At cellular levels of magnesium, the ATP binds first, followed by HMDP [23]; in the absence of cofactor and magnesium, HMDP binds weakly *in vitro* to the *apo* enzyme [24]. Both active sites are highly selective for their ligands. For example, the affinity of *E. coli* HPPK (EcHPPK) for Mg-GTP is 260-fold less than for Mg-ATP [25]. Remarkably, only two specific pterin-site inhibitors have been reported in the literature [26]. Both are based on the pterin substrate (Fig. 1), one featuring gem-dimethyl substitution at the C7 position on the pyrimidine ring, the other a phenethyl substituent at the same position. Bisubstrate analogues of the former have been reported that display sub-micromolar affinity, which demonstrates the feasibility of developing new inhibitors based on bisubstrate-linking strategies [27].

*S. aureus* HPPK (SaHPPK) shares 34–39% sequence homology with HPPK enzymes from other species whose structures have been determined (Fig. 2*C*). High conservation of active site residues, and high structural similarity among all HPPK structures, suggests that HPPK inhibitors developed for one species may have advantageous cross-reactivity over many different species.

Herein, we report the first structural studies of HPPK from *S. aureus* using a combination of solution NMR and x-ray crystallographic structure determination, and the identification of a novel pterin-site inhibitor 8-mercaptoguanine (Fig. 2*D*) by *in silico* ROCS screening (Rapid Overlay of Chemical Structures) and differential scanning fluorimetry (DSF) assay. The atomic structure of SaHPPK has been determined in complex with a new pterin-site inhibitor, revealing the molecular details of inhibitor association. Binding of the inhibitor, substrate and cofactor molecules were quantified using isothermal titration calorimetry (ITC) and surface plasmon resonance (SPR), while *in vitro* enzyme inhibition data was measured using a luciferase based luminescent assay. Detailed studies of ligand interactions using NMR highlight critical ligand-induced dynamic changes upon inhibitor, substrate and cofactor binding, which correlate with large entropic penalties to the binding thermodynamics of the inhibitor measured by ITC.

## Results

### Enzyme stability

Buffer conditions were screened to overcome initial solubility problems with SaHPPK using a differential scanning fluorimetry (DSF) assay [28] and button tests [29]. Final optimized buffer conditions from both techniques correlated well, with SaHPPK found to be most stable in a buffer comprised of 50 mM HEPES (pH 8.0), 1% sorbitol and 2.0–5 mM DTT.

### Validation of ROCS virtual screening hits using the DSF assay

To identify potential new binders of SaHPPK, we adopted a high-throughput virtual screening approach (see methods) using ROCS (Rapid Overlay of Chemical Structures) [30]. The DSF assay used for buffer stability screening was subsequently employed as an efficient method to screen the 37 purchased ROCS compound library. DSF is a rapid, convenient and inexpensive assay to detect binding of ligands to proteins. It compares the change in the unfolding transition temperature (*ΔT_m_*) of a protein obtained in the presence and absence of a ligand [31], [32]. A ligand that binds to the protein generally causes an increase in the protein thermal melting temperature (*T_m_*).

**Figure 3:**
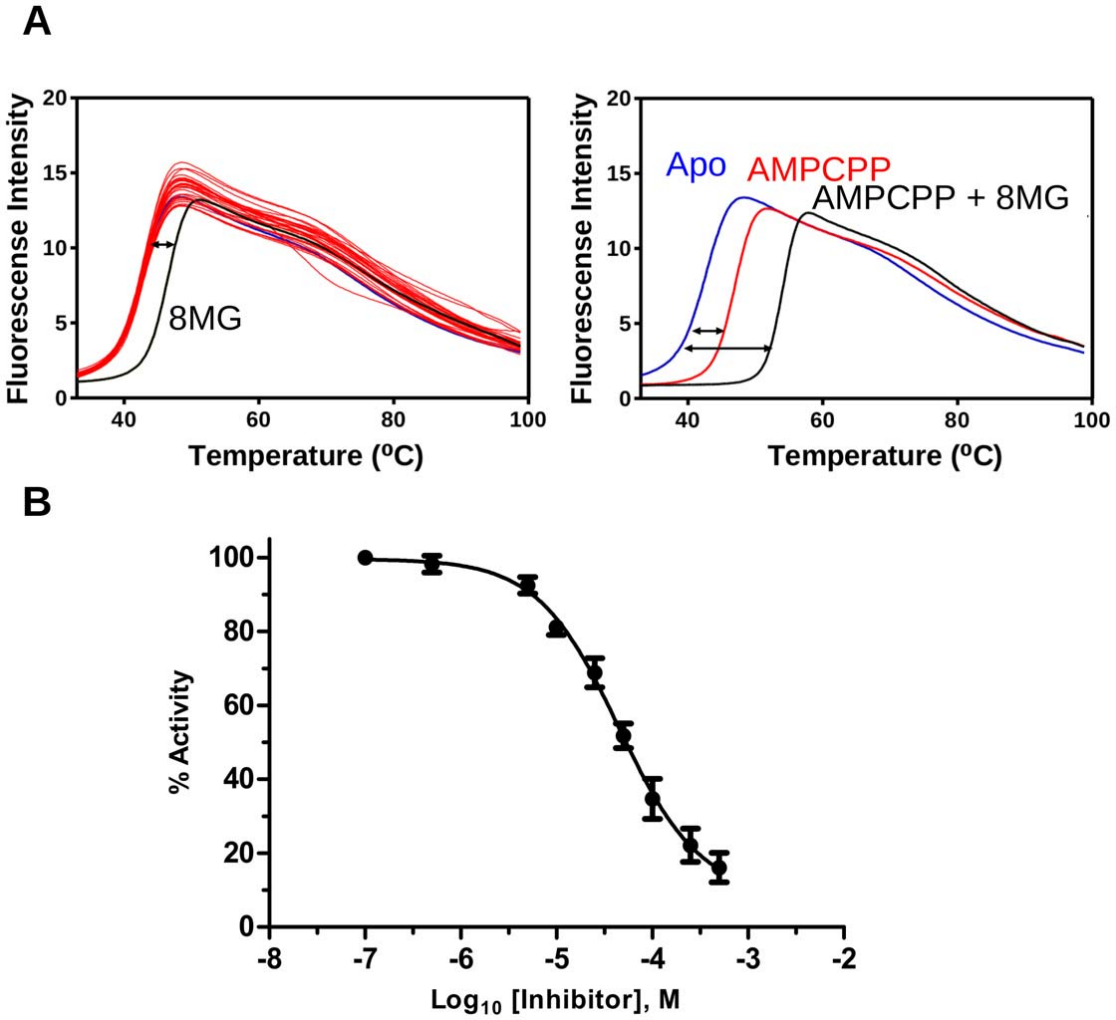
DSF and enzyme assays. A, Superposition of the DSF assay profiles of 37 ROCS compounds (*left*) showing the curve for 8-mercaptoguanine (8MG) (black) and thermal shift *ΔT*, double headed arrow) relative to the apo enzyme (blue) or relative to the AMPCPP complex (*right*). The DSF assay for AMPCPP is also shown (red). B, Dose response curves for the inhibition of SaHPPK by 8-mercaptoguanine (IC_50_ = 41 µM). HMDP and ATP concentrations were fixed at 0.3 µM and 0.2 µM respectively.

We tested the utility of the assay using ATP (and AMPCPP) binding. ATP and AMPCPP bind relatively tightly to EcHPPK, with dissociation constants of 2.6 and 0.45 µM, respectively [24]. The unfolding temperature (*T_m_*) of SaHPPK increased by 6 and 11°C in the presence of saturating ATP and saturating AMPCPP, respectively, which is consistent with the previously observed tighter binding of AMPCPP to EcHPPK, as well as the results of our ITC and SPR experiments (*vide infra*). Inspired by these encouraging results, this rapid assay was used to screen the ROCS-generated library, using a single ligand concentration of 500 µM in 5 µM enzyme. Out of the 37 compounds tested, one compound, 8-mercaptoguanine, produced an increase in *T_m_* of 3.4°C (as compared to *apo*), confirming binding to SaHPPK (Fig. 3*A*). Moreover, a similar increase in *T_m_* was observed in the presence or absence of ATP/AMPCPP, suggesting non-competitive binding with the cofactor.

### Biochemical assay

A KinaseGlo™ assay was performed to test SaHPPK function and examine whether 8-mercaptoguanine inhibits SaHPPK catalysis. While a K_m_^app^ value of 10.8±2.5 µM was readily obtained for ATP, the assay is insufficiently sensitive to allow reliable determination of the relatively low K_m_ for HMDP, consistent with those determined for the *E. coli* enzyme (0.7–1.6 µM) using different assays [23], [33]. The assay is however suitable to show inhibition and gave an apparent IC_50_ of 41±9 µM for 8-mercaptoguanine (Fig. 3*B*).

### Thermodynamics of ligand binding by ITC

**Figure 4:**
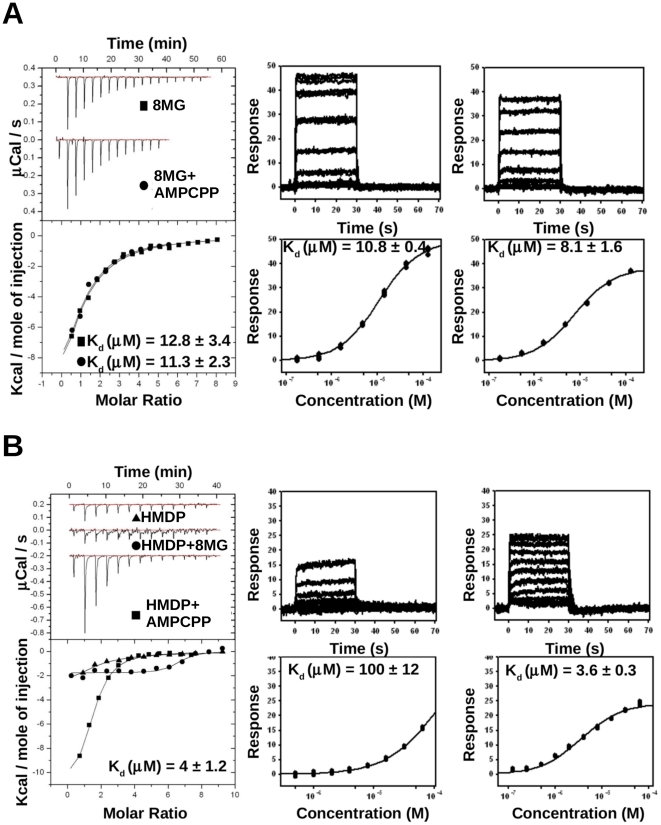
Binding of ligands to SaHPPK as measured by ITC and SPR. A, Raw (*top left*) and integrated ITC data (*bottom left*) for the titration of 30 µM SaHPPK with 300 µM 8-mercaptoguanine (8MG) alone (▪), and 300 µM 8-mercaptoguanine in the presence of 1 mM AMPCPP (•). SPR raw data (*middle* and *right top*) and steady-state response curve (*middle* and *right bottom*) for the binding of 8-mercaptoguanine in the absence (*middle*) and presence (*right*) of 5 mM ATP. B, Raw ITC data (*top left*) and integrated normalised data (*bottom left*) for titrations of 22 µM SaHPPK with 500 µM HMDP (▴) in the absence of 8-mercaptoguanine and in the presence of 300 µM 8-mercaptoguanine (•), and in the presence of 1 mM AMPCPP (▪). Binding of HMDP was only detected in the presence of AMPCPP. SPR raw data (*middle* and *right top*) and steady-state response curve (*middle* and *right bottom*) for the binding of HMDP in the absence or presence of saturated AMPCPP.

To determine the affinity and thermodynamic parameters of ligand binding to SaHPPK, we employed isothermal titration calorimetry (ITC) (Table 1; Fig. 4). Previous studies using fluorescence-based methods have reported a binding affinity (*K*_d_) of EcHPPK for ATP of 2.6–4.5 µM, and for AMPCPP of 0.08–0.45 µM in the presence of Mg^2+^ [23], [24], [25]. Binding affinities of EcHPPK for HMDP substrate vary from 0.036 to 0.17 µM in the presence of Mg^2+^ and AMPCPP [23], [24].

**Table 1:** Thermodynamic and kinetic parameters for the binding of ligands to SaHPPK, as determined by ITC^a^ and SPR^a^.

Ligand	Δ*H*(kCal mol^−1^)	*T*Δ*S*(kCal mol^−1^)	Δ*G*(kCal mol^−1^)	N	*K*_d_(µM) ITC	*K*_d_(µM) SPR
ATP	−6.5±0.4	−0.3±0.5	−6.2±0.1	0.98±0.11	31.0±4.5	45±2
ADP	nd^c^	nd^c^	nd^c^	nd^c^	nd^c^	760±16
AMP	nd^c^	nd^c^	nd^c^	nd^c^	nd^c^	4900±1100
AMPCPP	−6.3±1.0	1.3±1.0	−7.5±0.2	1.06±0.12	3.1±1.2	7.7±0.4
HMDP	nd^c^	nd^c^	nd^c^	nd^c^	nd^c^	100±12
HMDP+AMPCPP^b^	−10.5±1.0	−3.1±0.9	−7.4±0.2	1.23±0.15	4.0±1.2	3.6±0.3
8MG^e^	−19.6±3.4	−12.9±3.5	−6.7±0.2	1.00±0.06	12.8±3.4	10.8±0.4
8MG^e^+AMPCPP^b^	−17.2±1.4	−10.4±1.5	−6.8±0.1	0.96±0.09	11.3±2.3	nd
8MG^e^+ATP^d^	nd	nd^c^	nd^c^	nd^c^	nd^c^	8.1±1.6

a Values are the means ± the standard deviation for at least three experiments. See *Materials and Methods* for sample concentrations used. All ITC and SPR experiments were performed at 298 K and 293 K respectively.

b Concentration of AMPCPP was 1 mM in both HPPK (cell) and ligand (syringe) solutions.

c No data.

d Concentration of ATP was 5 mM.

e 8-mercaptoguanine.

ATP and AMPCPP were shown to bind exothermically to SaHPPK with affinities of 31 and 3.1 µM, respectively at 298 K (Table 1; Fig. S1 *A*). In the absence of Mg^2+^, no significant binding was observed for either compound (data not shown). These affinities are lower than those reported for EcHPPK, but we observe a very similar affinity ratio for the two compounds, with AMPCPP displaying approximately 10-fold tighter binding than ATP. The inhibitor was found to bind SaHPPK with a *K*_d_ of 12.8±3.4 µM (Fig. 4*A*), with AMPCPP having no effect on the affinity or thermodynamics of interaction. The interaction occurs with a large favourable binding enthalpy but with a significant entropic cost (see thermodynamic parameters in Table 1).

In saturating AMPCPP, the substrate (HMDP) bound with a *K*_d_ of 4.0±1.2 µM (Fig. 4*B*), which is again significantly weaker than that determined for EcHPPK by fluorescence methods [23], [24]. In the presence of the inhibitor 8-mercaptoguanine, however, binding was reduced to a level that could not be detected by ITC. Therefore, it appears that the inhibitor binds competitively to the same binding site as the substrate, affecting enzyme inhibition accordingly. In line with previous studies, we observed that HMDP binding is highly dependent upon the prior incorporation of ATP, or the analogue AMPCPP. Although a weak binding signal is observed for HMDP without AMPCPP, a satisfactory fit to the data could not be obtained, which is consistent with the low affinity estimated from SPR experiments (see below).

### Kinetics of ligand binding by SPR

Binding interactions of SaHPPK substrates, AMPCPP and 8-mercaptoguanine were analysed via surface plasmon resonance (SPR) using minimally biotinylated SaHPPK immobilized onto a NeutrAvidin chip surface (Table 1, Fig. 4 and Fig. S1). In this manner, we were able to obtain highly active SaHPPK surfaces not compromised by a low pH required for amine coupling method [34]. *K*_d_ values for ATP and AMPCPP were determined to be 45±2 and 7.7±0.4 µM, respectively, in close agreement with the values determined by ITC (Table 1, Fig. S1 *A*). The slower dissociation of AMPCPP also allowed the binding affinity to be derived by fitting to a kinetic interaction model, yielding rate and affinity parameters of *k*_a_ = 8.8±1.3×10^4^ M^−1^ s^−1^, *k*_d_ = 0.5±0.1 s^−1^ and a similar *K*_d_ = 5.4±0.3 µM (Fig. S1 *A*). Based on this result, we conclude that the higher affinity of AMPCPP compared to ATP is mainly driven by a slower dissociation rate parameter (*k*_d_) for AMPCPP. Weak binding of ADP and AMP (Table 1, Fig. S1 *B*), beyond the detection limit of ITC, could be estimated by steady-state fitting in SPR, extending the upper limit of detection. Binding of HMDP to SaHPPK in the absence of the cofactor was not readily detectable by ITC under the conditions used here, but could be approximated by SPR (*K*_d_∼100 µM). The binding affinity for the SaHPPK/HMDP interaction measured by SPR in the presence of AMPCPP (*K*_d_ = 3.6±0.3 µM) was in excellent agreement with the value obtained by ITC (*K*_d_ = 4.0±1.2 µM) (Table 1, Fig. 4*B*). Furthermore, SPR measurements confirmed that binding of 8-mercaptoguanine to SaHPPK was independent of cofactor, as estimated affinity values in the presence and absence of ATP were approximately the same (10.8±0.4 uM and 8.1±1.6 uM, respectively) (Table 1, Fig. 4*A*).

### Backbone assignments of SaHPPK with substrate and cofactor

The chemical shift of a nucleus is sensitive to changes in its local environment and is thus a convenient probe for analysing ligand binding events and detecting conformational changes. To investigate the structure and substrate binding properties of the enzyme, we thus assigned the backbone resonances of SaHPPK under various ligand conditions using heteronuclear NMR spectroscopy and compared the change in the weighted average resultant ^15^N and ^1^HN chemical shift vector (chemical shift perturbations, CSPs). All ^15^N HSQC spectra e.g. Fig. S2, showed well-dispersed sets of resonances, consistent with a folded enzyme.

No change over time was observed in the ^15^N HSQC spectra of the SaHPPK/HMDP/AMPCPP complex under the sample conditions (see methods), at least for a period up to ∼36–48 hrs (Fig. S3), which was long enough to record 3D experiments to make assignments and record relaxation experiments. Oxidation of HMDP has been reported in previous x-ray studies [22], however, while some degradation was observed in the 1D ^1^H of isolated HMDP alone over time (data not show), spectral evidence for a bound hydroxymethylpterin oxidation product or degradation species were not observed (Fig. S4). It appears that degradation over time leads to a weakly bound product which is not competitive to the tight binding substrate. Therefore, for the first time we report NMR data on the binding and dynamics of the substrate to HPPK.

Under physiological conditions, the SaHPPK enzyme is expected to be saturated with ATP, given the binding constant of ∼45 µM (Table 1, Fig. S1 *A*). Titration of SaHPPK with ATP or AMPCPP revealed slow exchange on the NMR timescale. Substantial CSPs were observed for both cases and large perturbations were localized to the ATP site (Fig. S2). A titration of SaHPPK with fresh HMDP to saturation levels showed extensive chemical shift broadening (or disappearance) compared to the *apo* ^15^N HSQC spectrum, and few residual CSPs. The broadening mapped to residues within loop L2 (Fig. S2 *B*, *C*) and residues around the pterin subsite, including those of the β-sheet, underneath the substrate. Adding HMDP to the saturated AMPCPP complex, on the other hand, revealed slow exchange, characteristic of tighter binding. Perturbations clearly mapped to the pterin site, as expected (Fig. S2 *B*, *C*) yet a few resonances in loop L2 still remained broadened.

Given that the inhibitor has no non-exchangeable ^1^H NMR signals and standard NMR techniques would only poorly characterise the intermolecular interactions, we adopted a parallel investigation of the x-ray structure in complex with 8-mercaptoguanine.

### X-ray crystallographic structure of SaHPPK in complex with 8-mercaptoguanine at 1.65 Å resolution

**Figure 5:**
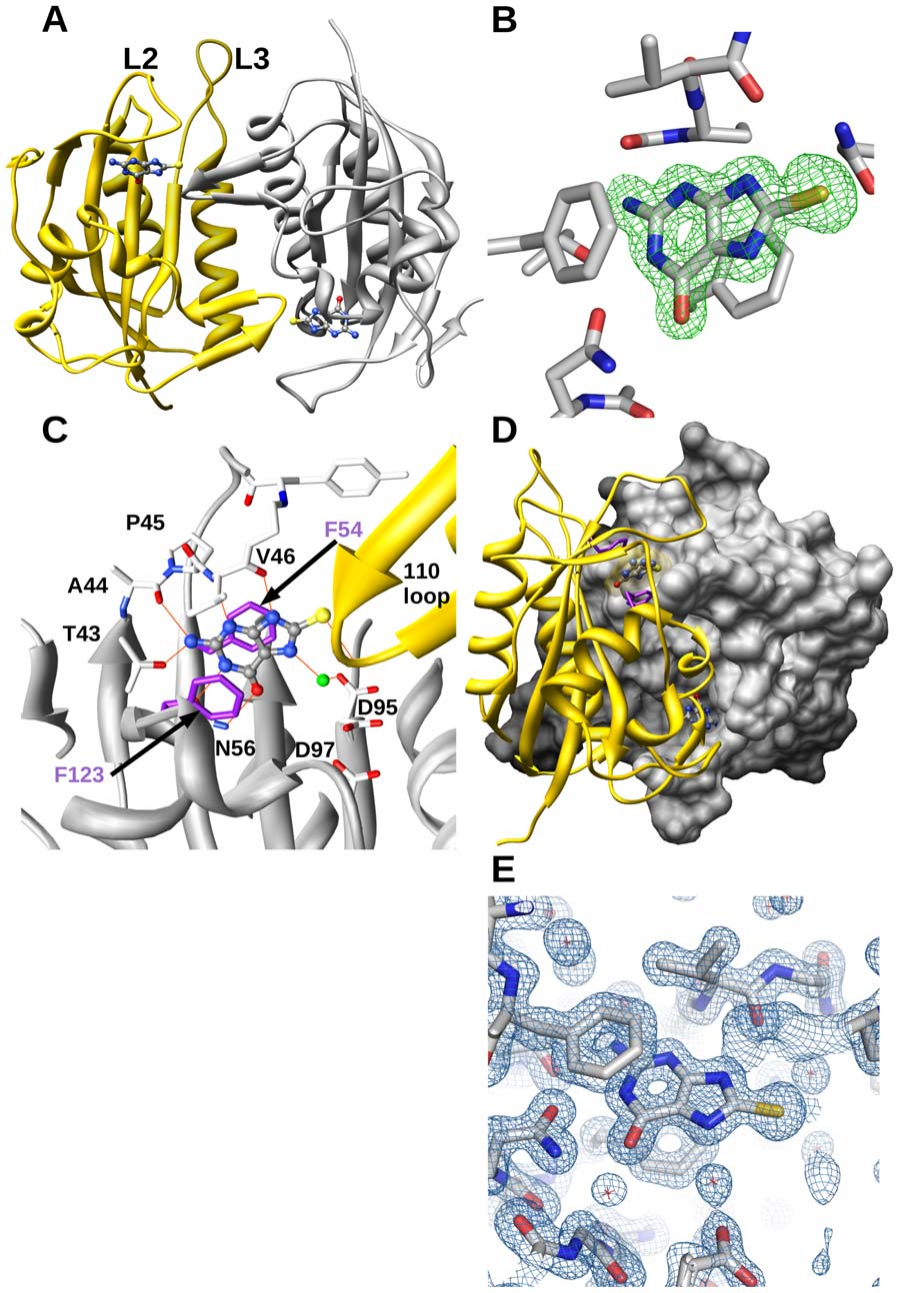
X-ray structure of SaHPPK with the 8-mercaptoguanine inhibitor bound. A Ribbon schematic showing the crystallographic dimer of theSaHPPK-8-mercaptoguanine binary complex. B, F_0_-F_c_ difference density map of 8-mercaptoguanine contoured at 3.0 sigma. C, Intermolecular interactions (orange) involving 8-mercaptoguanine. Two side chain conformers for Asp95 were modelled and are shown. The second monomer is drawn in yellow ribbon. The oxygen atom of a water molecule forming hydrogen-bonds with 8-mercaptoguanine and SaHPPK is shown in green. D, Space filling representation of the dimer interface, showing 8-mercaptoguanine bound in the deep pterin pocket, sandwiched between Phe123 and Phe54 (purple), and illustrating the proximity to the 110 s loop in the other monomer. E, Final 2F_0_-F_c_ electron density map of the pterin binding site, contoured at 2.0 sigma.

The x-ray crystal structure of SaHPPK in complex with the inhibitor, 8-mercaptoguanine was solved at high resolution (Fig. 5). Crystallisation conditions were as published [35]. Briefly, the SaHPPK/8-mercaptoguanine binary complex crystallised in the P2_1_ space group, with the asymmetric unit comprising two protein molecules that contain a single bound 8-mercaptoguanine molecule per monomer and a total of 256 water molecules (Fig. 5*A*). Density was observed for all 158 amino acid residues of the protein, although the density for residues 85–91 was very weak in monomer B. Two (in chain A) or three (in chain B) additional non-native residues are seen as a result of the *N*-terminal thrombin cleavage site.

SaHPPK has a ferredoxin-like fold (αβα), with a central core of six β strands surrounded by four helices, typical of other monofunctional HPPK structures. The two monomers in the asymmetric unit are almost identical, with an RMSD of 0.34 Å over all 158 pairs of backbone Cα atoms. The catalytic loops L1, L2 and L3 (residues 12–14, 47–51, 82–94) have higher than average temperature factors, showing that they are likely mobile in solution, which parallels the observed broadening of residues in solution by NMR. The dimer interface encapsulates the active sites, leading to a buried surface area of 1595 Å, not including the inhibitor molecules. Eight inter-subunit hydrogen bonds are observed.

Using the SSM algorithm in Coot [36], we calculated 36% sequence identity between the structures of SaHPPK and EcHPPK (1RAO), with a RMSD of 1.45 Å over the 148 aligned residues. While the structure of SaHPPK deviates significantly from the EcHPPK in the region leading out to the C terminus (residues 150–156) (Fig. 2*B*), secondary structural elements are generally very well conserved as they are across all HPPK enzymes (Fig. 2*B*), with major variations occurring mostly in the catalytic loops, dependant on the types of bound ligands or catalytic stage.

### Comparison of 8-mercaptoguanine and HMDP binding

The crystal structure confirms that 8-mercaptoguanine binds to the pterin pocket in a similar pose to the HMDP substrate bound in EcHPPK (1Q0N) (Fig. 5*B, C*). Mercaptoguanine and HMDP share the same pyrimidine ring, which in both cases makes hydrogen bond contacts with several highly conserved residues; T43, A44, V46, and N56 saturate the hydrogen bond donor/acceptor sites (positions 1, 2, 3, 4, and 9) of the inhibitor (Fig. 5*C*). Like HMDP, the pyrimidine ring of 8-mercaptoguanine is stacked between the conserved aromatic residues, Phe54 and Phe123. A small cavity is found near the N7 position of 8-mercaptoguanine, in which a single water molecule resides, forming hydrogen bonds with the sidechain of Asp95 and the N7 of 8-mercaptoguanine. Notably, two of the interactions formed between HMDP and HPPK are absent in the case of 8-mercaptoguanine. Firstly, the imidazole ring of 8-mercaptoguanine is too far away to interact with Asp95, which forms a hydrogen bond with the hydroxymethyl group of HMDP. Secondly, a van der Waals interaction with Trp89 is missing. Loop L3 is displaced out of the active site and the Trp89-8-mercaptoguanine distance is around 25 Å, compared to ∼4 Å in the HMDP/AMPCPP-bound structure of EcHPPK (pdb 1Q0N).

### Comparison of loop positions

The three loops of HPPK (L1–L3) are highly dynamic in nature, changing conformation during the enzymatic cycle (Fig. 2*B*) [37]. Loops L1 and L2 undergo relatively minor structural changes compared to loop L3, for which the apex moves over 20 Å throughout the cycle (Fig. 2*B*). Loop L2 in SaHPPK caps the substrate active site and resembles closely the loops in the HMDP/AMPCPP (1Q0N) and pterin analogue-bound forms (1DY3, 1CBK). Loop L3 in SaHPPK is “extended” out from the active site and, as such, resembles most closely the loop position in the product-bound EcHPPK structure (1RAO). It is excluded from the active site by the 108–111 loop from the other monomer in the asymmetric unit (Fig. 5*C, D*).

Although we grew crystals routinely in the presence of 2 mM AMPCPP and 10 mM Mg^2+^, we did not observe density for either, even when the concentrations of these species were increased to 25 and 50 mM, respectively. We therefore performed NMR measurements on the ternary SaHPPK/8-mercaptoguanine/AMPCPP complex.

### Chemical shift mapping of inhibitor complexes by NMR spectroscopy

**Figure 6:**
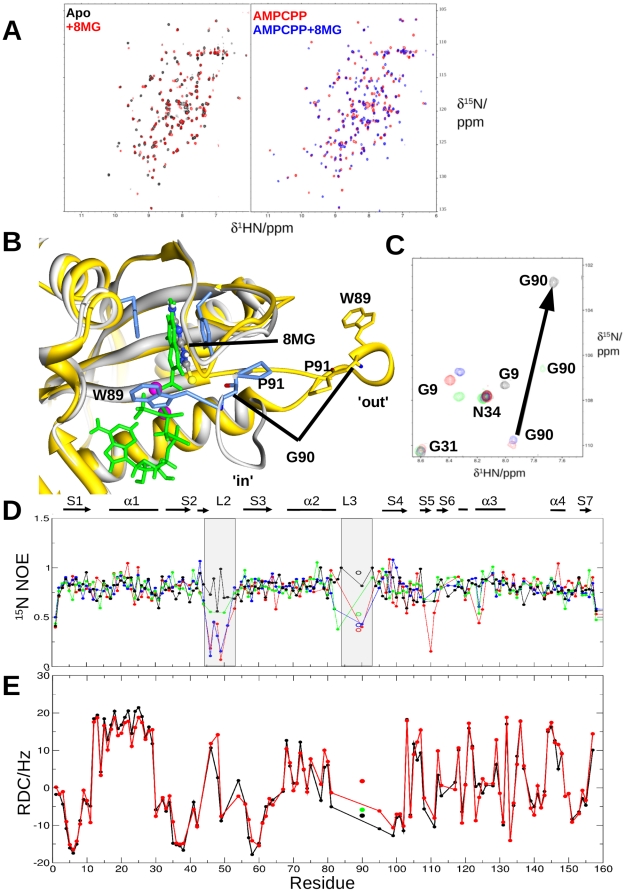
Binding of 8-mercaptoguanine (8 MG) to SaHPPK by NMR spectroscopy reveals an ‘in’ conformation of loop L3. A, Superposition of the ^15^N HSQC spectra of ∼100 µM SaHPPK with and without 0.6 mM 8-mercaptoguanine (*left*), and the ^15^N HSQC spectra of ∼100 µM SaHPPK+1 mM AMPCPP+10 mM Mg^2+^ with and without the addition of 600 µM 8-mercaptoguanine. B, Superposition of the EcHPPK/AMPCPP/HMDP (1Q0N, grey ribbon, blue sidechain, green ligands) with SaHPPK (yellow). The loop L3 ‘in’ conformation and the loop ‘out’ conformation are shown for each respectively and the sidechain of Trp89 and Pro91. C, Region of the superposed ^15^N HSQC spectra of *apo*SaHPPK (red), SaHPPK/ATP complex (blue), SaHPPK/HMDP/AMPCPP complex (green) and SaHPPK/8-mercaptoguanine/AMPCPP complex (black). The large CSP of G90 is shown. D, Superposition of the ^15^N heteronuclear NOE values recorded at 600 MHz. The colour coding is the same as in C). Open circles are for the Hε1 of Trp89. The extent of the secondary structural elements is shown at the top, with the region corresponding to the active site loops, L2 and L3, highlighted. E, Comparison of observed (black) and calculated (red) ^1^D_HN_ RDCs for SaHPPK/8-mercaptoguanine/AMPCPP. The ^1^D_HN_ RDC for G90 from EcHPPK/HMDP/AMPCPP (1Q0N) is shown (green filled circle).

Titration of 8-mercaptoguanine into a sample of the *apo* enzyme produced a range of CSPs (Fig. 6*A*) and exchange regimes in the NMR spectra. 15 cross peaks broadened completely around the binding site and along the sheet (Fig. S2 *D*) and most others exhibited slow exchange, indicative of a *K*_d_ likely in the low µM range. In contrast, when performed in the presence of saturating ATP or AMPCPP, widespread perturbations were observed in slow exchange for all resonances, despite no change in binding affinity measured by ITC and SPR. Chemical shift perturbations clearly mapped to the respective substrate and cofactor site (Fig. S2 *D*).

All ^15^N and ^1^HN amide chemical shifts are tabulated in Table. S1 and values for CSPs are shown in Fig. S5.

### SaHPPK is a monomer in solution

The ^15^N T1/T2 ratio for amides of SaHPPK in the presence of 8-mercaptoguanine and AMPCPP correlated well with that calculated using HYDRONMR [38] for a monomeric enzyme with a correlation time of ∼12 ns (Fig. S6). This clearly showed that the enzyme exists as a monomer in solution at a concentration less than ∼200 µM. Line widths were similar for the SaHPPK/8-mercaptoguanine binary complex.

### The linewidth and chemical shift of Gly90 amide indicates a loop ordering in the vicinity of the inhibitorfor the inhibited ternary complex

We observed that the amide of Gly90 displayed a large upfield CSP, and sharpened significantly, when comparing the spectrum of the SaHPPK/AMPCPP complex to that of the SaHPPK/8-mercaptoguanine/AMPCPP complex (Fig. 6*C*). In addition, a similar but smaller shift was observed in the SaHPPK/HMDP/AMPCPP complex and Gly90 was noticeably broadened (Fig. 6*C*). Such CSP reflects a dramatic change in the environment and a decrease in local chemical exchange effects respectively, likely associated with a decrease in the motion of loop L3 on the µs-ms timescale. Further evidence for a decrease in loop L3 dynamics is supported by more extensive assignments in loop L3 for the SaHPPK/8-mercaptoguanine/AMPCPP spectra relative to all other spectra (compare the ribbon diagrams in Fig. S2 *B* with Fig. S2D).

### ^15^N fast timescale dynamics uncovers distinct loop dampening in the inhibited ternary complex

To understand the fast timescale motion of SaHPPK in solution we recorded ^15^N heteronuclear NOE data for the *apo* enzyme, the binary SaHPPK/ATP complex and the SaHPPK/8-mercaptoguanine/AMPCPP ternary complexes (Fig. 6*D*). Data for the 8-mercaptoguanine or HMDP binary complexes were not recorded owing to extensive broadening around the substrate site.

In all cases, the last and first residues showed relatively low ^15^N NOE values (∼0.4–0.6) and are therefore partially mobile on the ps-ns timescale. The amides of Val46–Gln51 in loop L2 were also mobile for the ATP-bound and *apo* samples. Gly90 was found to be mobile on fast timescale in *apo* and ATP-bound SaHPPK. Val124, the residue adjacent in sequence to the ring-stacking Phe123, appeared to be mobile on the ps–ns timescale (NOE∼0.6) with all substrates.While Lys110 is mobile in the apo enzyme signal overlap precluded assessment of this residue in the complexes.

For the ternary SaHPPK/8-mercaptoguanine/AMPCPP complex, apart from Tyr48, the fast motions within loop L2 diminish and the loop essentially becomes rigid around the inhibitor (Fig. 6D). This is in contrast to the SaHPPK/HMDP/AMPCPP complex which remains partially mobile. While the fast motions of Gly90 and the sidechain Hε1 of Trp89 are considerably dampened in the inhibited ternary complex compared to all other complexes, there is evidence for slower underlying motion particularly in the 84–88 region as judged by resonance broadening. Finally, the fast timescale motion that was evident in Val124 (^15^N NOE∼0.5) in all other spectra also appears to dampen and this amide becomes rigid in the SaHPPK/8-mercaptoguanine/AMPCPP complex.

### ^15^N Residual dipolar couplings (RDCs) support a closed loop L3 conformation for the inhibited ternary complex in solution

To understand the structure of the inhibited ternary complex in solution we recorded ^1^D_HN_ residual dipolar couplings (RDCs) of each amide. These are induced by the weak alignment of a biomolecule in solution and report the angle a given N-H vector makes within the principal axis system of the alignment tensor. Weak alignment of SaHPPK was achieved with a PEG/hexanol alignment media [39] from which RDCs up to ∼20 Hz in magnitude were measured (see methods). These allowed us to derive the alignment tensor by single value decomposition within PALES [40] by fitting the measured ^1^D_HN_^15^N RDCs to our x-ray structure (Fig. 6E). The striking agreement (Pearson's correlation coefficient (R^2^) = 0.97, Q factor = 24%) between the ternary SaHPPK/8-mercaptoguanine/AMPCPP complex in solution with the binary SaHPPK/8-mercaptoguanine x-ray structure shows that, overall the binary structure is a very good model for the ternary structure in solution. Similarly oriented amides within helix 1 for example (residues 14–28) lie parallel to the principal axis of the alignment tensor as evidenced by a string of fairly uniform RDCs with maximal value. Helix 2 on the other hand shows smaller RDCs and larger sinusoidal fluctuations as it is tilted away from the principal axis.

A few RDCs stand out as outliers. Interestingly, these generally map to those amides in close proximity to a ligand or metal. Residues 123–125 showed very small RDCs (0.38, 1.0, and −1.42 Hz respectively) and their calculated values (−8.7, −3.8, 4.8 Hz) result in statistically significant deviations (>∼5 Hz). Low values of the RDC can result from motional averaging of the amide vector or if the NH vector points close to the magic angle with respect to the Z axis of the alignment tensor. As the ^15^N NOE values are high for residues 123–125, the RDC data suggest small tilts of these amide vectors in solution towards the magic angle compared to those of the x-ray structure. Calculations show that rotations of 18, 36 and 22° giving small displacements of 0.3, 0.6 and 0.4 Å respectively in the amide proton coordinate can accommodate these observed RDC values.

The ^1^D_HN_ RDC for Tyr48 (2.7 Hz) deviates from the calculated (14.2 Hz) and given the ^15^N NOE∼0.55 is most likely due to motion. The measured RDC for Asp95 (−10.9 Hz) deviated markedly from that calculated from the SaHPPK/8-mercaptoguanine x-ray structure (−3.3 Hz), but agreed very well to the amide orientation calculated (−12.3 Hz) for the EcHPPK/HMDP/AMPCPP ternary structure (1Q0N) indicating that a ∼30° reorientation of its H-N vectoris likely in the SaHPPK/AMPCPP/8-mercaptoguanine ternary complex. Interestingly, for the Gly90 amide in loop L3, the agreement with our x-ray structure is very poor indeed and of opposite sign, deviating by over 9 Hz. However, there is a close agreement (1.5 Hz) with the EcHPPK/HMDP/AMPCPP (1Q0N) orientation (Fig. 6E).

## Discussion

This work reports the discovery, binding properties and mechanism of a novel, competitive pterin site inhibitor, presented in complex with the first crystal structure of SaHPPK. The pterin site is highly specific and restricts the chemical space available for inhibitor design to structures closely resembling the pterin scaffold. Consequently, the literature is devoid of non-pterin like HPPK inhibitors [41], despite mounting structural information that has been reported over the last decade. In line with the high pterin-site specificity is the high ligand efficiency (2.3 kJ/heavy atom or*K*_d_∼13 µM over 12 heavy atoms) of 8-mercaptoguanine.

8-Mercaptoguanine has previously been reported to have biological activity. Early studies revealed some lipolytic activity [42] while in a number of cases 8-mercaptoguanine has been shown to inhibit enzymes that normally bind purines [43], [44], [45], [46]. Antiviral activity,without significant toxicity, was also reported in an *in vivo* mouse model [43]. Close analogues, such as 8-mercaptoguanosine, were also shown to induce interleukin-1 activity in macrophages [47]. Despite these studies, no antibacterial activity has been reported previously. Interestingly, 8-mercaptoguanine has been shown to bind to, but not inhibit, *B. anthracis* DHPS by co-crystallisation [48], which may open the possibility for a multi target inhibitor derived from this scaffold. In the present work, we did not observe growth inhibition *in vivo* by 8-mercaptoguanine in *E. coli* cell-based assays (data not shown). Given the unfavourable logP (−0.39), this is likely to be due to poor membrane permeability. This may be a disadvantage for pterin-like inhibitors in general given the hydrophilic nature and restrictive chemical space of the pterin scaffold in folate pathway enzymes. Nevertheless, while insufficient transport of a set of closely related pyrimidines as potential antifolates was implicated in their poor *in vivo* inhibition, derivatives with an additional phenyl substituent displayed sub micromolar activity *in vivo* to *T. brucei* and *L. major* [49]. The known phenethyl *in vitro* inhibitor of HPPK (pdb 1DY3) suggests that a suitably positioned phenyl group on 8-mercaptoguanine may thus be beneficial to both binding and assist cell permeability.

Given that 8-mercaptoguanine forms exactly the same number of inter-molecular hydrogen bonds as the substrate heterocyclic rings, an intriguing finding from our work is that, in the absence of cofactor, the inhibitor binds some 10-times more tightly (*K*_d_∼12 µM) than HMDP (*K*_d_∼100 µM by SPR). In the HMDP/AMPCPP complex the higher affinity (*K_d_*∼4 µM) of substrate can be rationalised by the observation of a hydrogen bond to the Mg^2+^ bound γ-phosphate of AMPCPP from the HMDP hydroxyl as seen in the EcHPPK/HMDP/AMPCPP (1Q0N) ternary complex [21], [41]. We therefore hypothesised that the large increase in the Δ*G* of binding might be associated directly with the sulfur atom in 8-mercaptoguanine, in the absence of substantial de-solvation or structural differences. While our SaHPPK structure is a valuable resource and will support further inhibitor design strategies towards the phosphate sub-site, the ‘out’ position of loop L3 in our crystallographic dimer precluded us from drawing many conclusions about the nature of the interaction between the sulfur atom of the inhibitor and enzyme. Nevertheless, we have established from several different types of NMR data that residue Gly90 is in close contact with the sulfur atom of the inhibitor in solution (Fig. 6).

In the EcHPPK/HMDP/AMPCPP ternary complex (1Q0N), the tip of loop L3 is observed closed ‘in’ over the active site with the Trp89 sidechain Hε1 hydrogen bonded to the terminal phosphate. A superposition of this ternary complex with our crystal structure indicates that the sulfur atom of the inhibitor would lie ∼4.6 Å from the sidechain of Trp89 and only ∼3.9 Å from the N atom of Gly90 (Fig. 6*B*). The importance of this proximity is likely to account for a specific interaction to the inhibitor and thereby stabilizing loop L3 into the ‘in’ position. For this loop arrangement and in a protonated thiol tautomer of the inhibitor, a hydrogen bond is predicted between the thiol proton and the backbone carbonyl of Gly90. The large ^15^N chemical shift perturbation (∼7 ppm),^15^N spin relaxation data and NMR linewidth considerations (Fig. 6) is evidence that the Gly90 amide is in close proximity and, along with the sidechain of Trp89, becomes essentially rigid in the ternary SaHPPK/inhibitor/AMPCPP complex. A direct interaction with the inhibitor is not possible from a solvent exposed Gly90/Trp89 in a loop L3 ‘open’ arrangement. In agreement with an important role of the sulfur atom of 8-mercaptoguanine in HPPK binding, guanine, the sulfur-free analogue of the inhibitor, displays markedly reduced affinity that was undetectable by SPR (data not shown) and gave few CSPs in the NMR spectra (data not shown). Finally, the close agreement of the ^15^N RDC for Gly90 to the EcHPPK ternary complex structure (1Q0N) (Fig. 6*E*) is evidence that G90 amide is oriented in solution as that in 1Q0N and thus further evidence of the loop ‘in’ conformation.

Active site motions and associated changes in loop conformations are an intrinsic requirement for enzyme activity [50] and for the structural transitions along the catalytic pathway of HPPK [51]. We propose that the thiol-mediated stabilization of loop L3 in turn stabilizes interactions to the neighbouring substrate loop L2. ITC data is in agreement with ^15^N relaxation data and showed a significant-binding entropy penalty for 8-mercaptoguanine binding to the SaHPPK/AMPCPP complex supporting the contention that the loops L2 and L3 (particularly around Gly90) become more ordered. In contrast, the lower entropic penalty for the binding of the substrate to SaHPPK/AMPCPP is in accord with a partially mobile loop L2 and loop L3 which is likely to be functionally relevant to facilitate subtle structural changes, during pyrophosphoryl transfer. Inhibition by 8-mercaptoguanine therefore may be derived in part from impeding a catalytic role of loop L3.This notion is consistent with the observation that the loop L3 deletion in EcHPPK did not affect ATP affinity, but produced a 10^5^-fold decrease in the rate constant for catalysis [20].

Intermolecular interactions to loop regions, may compromise an inhibitor as a potential long-term antimicrobial candidate. It is well documented that point mutations in loop regions have resulted in rapid sulfa drug resistance in the downstream DHPS enzyme [12], [48]. Given the C8 thiol of 8-mercaptoguanine seems to make an important backbone, rather than sidechain interaction with the highly conserved Gly90 or other natively small residue substitutions (Ala or Ser) in other species, (Fig. 2), this portion of the molecule may be a beneficial component of the pharmacophore in future chemical elaboration.

Over the last decade there has been a growing interest in using RDC calculations as a powerful additional parameter for the validation and refinement of macromolecular structures [52], [53]. Recently, chemical shift changes in conjunction with ^1^D_HN_ RDC measurements have revealed ligand induced conformational changes in the active site loop in ABL kinase [54]. Here, we have established unequivocally that the SaHPPK/8-mercaptoguanine/AMPCPP ternary structure in solution is essentially the same as the x-ray structure of the binary inhibited complex (for all residues that we could measure ^1^D_HN_ RDCs) and that loop L3 is likely ‘in’ (Fig. 6E). Furthermore, several NOEs to the H2 and H8 of AMPCPP were observed from amides 98, 111 and 112 in the 3D ^15^N NOESY HSQC experiment, consistent with the pose of the adenine found in the EcHPPK structure (1Q0N) (data not shown). These data show that our structure is also a good working model of the adenine-binding pocket.

In summary, our multi-disciplinary study shows that 8-mercaptoguanine readily binds to SaHPPK with high ligand efficiency and dampens loop motions by making specific interactions to both catalytic loops. It inhibits enzyme function *in vitro* and thus presents as an important scaffold for development of SaHPPK inhibitors with increased potency and more favourable pharmacokinetic properties. Conservation of the binding site within HPPK enzymes from *E. coli*, *H. influenza*, *Y. pestis*, *S. cerevisiae* and *F. tularensis* indicates that 8-mercaptoguanine may also be active against a range of other bacterial and lower eukaryotic enzymes. Accordingly, 8-mercaptoguanine may present a novel scaffold for future broad spectrum antibiotic development in the treatment of *S. aureus* and other pathogenic infections. We are currently exploring elaboration of the 8-mercaptoguanine scaffold to this end.

## Methods

### Crystallisation and x-ray structure determination

SaHPPK was expressed, purified and crystallised as described previously [35]. The initial phases of the binary complex were determined by molecular replacement using Phaser [55] as reported [35]. Prior to molecular-replacement calculations, the ligands (AMP, HMDPPP) and solvent molecules were omitted. Refinement was performed using *REFMAC*5 [56] and the Fourier maps *(2F_o_−F_c_ and F_o_−F_c_)* were visualized in *Coot* [57]. After several rounds of manual rebuilding, 8-mercaptoguanine and water molecules were added and the model further refined to a resolution of 1.65 Å. Structure validation was conducted using *PROCHECK* [58]. Table 2 provides the statistics for the x-ray data collection and final refined model.

**Table 2:** X-ray structure data processing and refinement statistics.

Spacegroup	Monoclinic, *P*21
x-ray source	MX2, Australian Synchrotron
Detector	ADSC Quantum 315
Wavelength (Å)	13000 eV (0.96Å)
Unit-cell parameters (Å, °)	a = 36.8, b = 76.6, c = 51.5 90, α = γ = 90.0, β = 100.2,
Diffraction data	
Resolution range (Å)	50.70-1.65 (1.74-1.65)
No. of unique reflections	33864 (4934)
No. of observed reflections	242194
Matthews coefficient, *V*_M_ (Å^3^ Da^−1^)	2.04
Solvent content (%)	39.6
Completeness (%)	100 (100)
Data redundancy	7.2 (7.2)
Mean I/σ(I)	18.3 (4.7)
R_merge_ (%)*	10.22(0.447)
R_p.i.m._ (%)#	4.1 (17.8)
Refinement (42.3–1.65 Å)	
*R*_free_ (%)	22.5
*R*_cryst_ (%)	17.9
Size of *R*_free_ set (%)	5
Protein native residues (dimer)	316
8-MERCAPTOGUANINE Molecules	2
Water molecules	254
*RMSD from ideal values:*	
Bond lengths (Å)	0.024
Bond angles (°)	2.16
Mean B factors (Å^2^)	14.7
Ramachandran plot	
Residues in most favored regions (%)	91.6
Residues in additionally allowed regions (%)	8.1
Residues in generously allowed regions (%)	0.4
Residues in disallowed regions (%)	0.0

*R_merge_ = ΣhΣi |*I*i(h)−<*I*(h)>|/ΣhΣi*I*i (h),

# R_pim_ = Σh [1/(N-1)]1/2 Σi |*I*i(h)−<*I*(h)>|/ΣhΣi*I*i (h).

Values in parentheses refer to the outer resolution shell (1.74-1.65Å).

Where *I* is the observed intensity, <*I*> is the average intensity of multiple observations from symmetry-related reflections, and N is redundancy.

R_value_ = _jjFoj _ jFcjj/_jFoj, where Fo and Fc are the observed and calculated structure factors. For R_free_ the sum is done on the test set reflections (5% of total reflections), for R_work_ on the remaining reflections.

### Preparation of isotopically-labelled protein for NMR spectroscopy

Isotopically-labelled protein samples for NMR spectroscopy were prepared as follows: *E. coli* BL21 (DE3) cells (Agilent) transformed with the plasmid were grown overnight in 3 mL of 2×YT media supplemented with 100 µg mL^−1^ kanamycin for selection. The overnight culture was subcultured into 50 mL of minimal media that was grown to an OD_600_ of 0.5–0.7. This was then added to 1 L of minimal media supplemented with 1.5 g of ^15^N ammonium chloride and 3 g of ^13^C glucose and grown at 310 K until the OD_600_ was 0.5–0.8. Isopropyl β-D-1-thiogalactopyranoside (IPTG) was added to a final optimised concentration of 0.5 mM and expression was carried out at 293 K for 12 hr. Purification was as reported previously [35].

### NMR spectroscopy

All NMR experiments were recorded at 295 K on a Varian Inova 600 MHz NMR spectrometer equipped with a cryoprobe and Z axis gradient. Triple resonance assignments were performed on SaHPPK, firstly in the presence of 10 mM Mg^2+^ and 1 mM AMPCPP, and secondly with the further addition of 0.6 mM 8-mercaptoguanine. ^15^N/^13^C-labelled SaHPPK was typically 0.15–0.25 mM in NMR buffer (90%/10% H_2_O/D_2_O buffer of 50 mM HEPES pH 8.0, 1% sorbitol, 10 mM DTT). Backbone assignments were obtained on these samples using the following triple resonance experiments: HNCO, HNCA, HN(CO)CA, HNCACB, CBCA(CO)NH. Assignments were further confirmed using a 3D ^15^N-edited NOESY experiment recorded with a mixing time of 120 ms [59]. The ^15^N ^1^HN assignments of the following samples were derived from a pair of 3D experiments; a ^15^N edited NOESY experiment recorded with a mixing time of 120 ms and a HNCA experiment: *apo*SaHPPK, SaHPPK in the presence of 0.6 mM 8-mercaptoguanine, and SaHPPK in the presence of 1 mM HMDP/10 mM Mg^2+^/1 mM AMPCPP in NMR buffer. Titrations were performed by titrating ligands into 0.1 mM^15^N-labelled SaHPPK protein samples and recording a soFast^15^N HMQC spectrum [60].

HMDP is prone to oxidation and degradation [22] which has complicated x-ray or NMR studies to date. No change was observed in the protein spectra over the time-course of all NMR experiments (<48 hrs, Fig. S3). Samples were routinely sealed under nitrogen for all lengthy NMR experiments to slow disulfide mediated aggregation or air/light induced degradation of HMDP.

^15^N relaxation data were recorded on ∼0.15 mM ^15^N-labelled samples of SaHPPK. ^15^N heteronuclear NOE spectra were recorded using TROSY-type selection and with watergate suppression [61], owing to superior sensitivity compared to the sensitivity-enhanced version [62] on the Varian cryoprobe. Three seconds of weak presaturation was used to generate the desired heteronuclear NOE, and was applied on- or off-resonance at the amide proton frequency, in addition to 1 s of relaxation delay. T1 and T2 relaxation data were acquired as described [62]. The relaxation delay was sampled at 10, 30, 60, 90, 110, 200, 500, 600 ms, and 10, 30, 50, 70, 90, 110 ms for longitudinal and transverse relaxation measurements, respectively. Spectra were processed using nmrPipe [63] and analysed with XEASY [64] or SPARKY [65].Titration and relaxation 2D experiments were acquired with t_1max_ (^15^N) = 51–62 ms and t_2max_(^1^H) = 142 ms.

^1^D_NH_ RDCs were measured on a ∼0.1 mM ^15^N-labelled SaHPPK sample in the presence of an anisotropic media comprising 5% (wt/vol) C12E6/hexanol [39]. RDCs were obtained by comparing coupled spectra in the presence of the orienting media against spectra in the isotropic state by recording a 2D ^15^N IPAP-HSQC spectrum [66]. RDCs were measured using SPARKY as the isotropic (J) – aligned (J+^1^D_NH_) values recorded in the ^15^N (t1) dimension. 114 ^1^D_NH_ RDCs were fitted to the x-ray structure using single value decomposition incorporated into the “bestFit” module within PALES [40]. The ^1^D_NH_ RDCs were removed for couplings derived from severely overlapping peaks in the 2D IPAP spectra and mobile residues as inferred from ^15^N relaxation data. The error in the RDC was conservatively estimated as +/− 1 Hz, according to the ratio of the linewidth to the signal-to-noise. Final values of Da and Rhombicity were 9.6 Hz and 0.53 respectively.

### Virtual screening using ROCS (Rapid Overlay of Chemical Structures)

An *in-silico* database comprised of 229,172 commercially purchasable compounds (Maybridge, Chembridge and Specs libraries) was used for the screening. Database compounds were first converted from 2D sdf format into 3D coordinates using LigPrep within Maestro [67]. A ROCS [68] run was then initiated to screen the database for potential pterin site binders using HMDP as the query molecule. The top 500 hits were ranked according to combined volume overlap (Tversky) and chemical functionality (Colour) scores. The hits were overlayed onto the crystal structure of EcHPPK bound to HMDP (1Q0N). Filtering of incompatible molecules was first assessed by manual inspection of the hits within the pterin binding pocket and compounds that showed any possible steric clashes were discarded. ROCS hits were chosen that retained key pharmacophore interactions in the pterin binding site and those that provided synthetic opportunities for scaffold optimization. A total of 44 compounds were selected and of these, 37 were available for purchase.

### Differential Scanning Fluorimetry (DSF) assay for ligand binding

A 96-well plate containing solutions of 2–5 µM SaHPPK, 10× Sypro orange dye and 500 µM of test compound in buffer (50 mM HEPES (pH 8.0), 1% sorbitol, 2.0 mM DTT, 10 mM MgCl_2_) was heated from 298 to 322 K at a rate of 1.0 K min^−1^. The fluorescence intensity was measured with excitation/emission wavelengths of 583/610 nm. *T_m_* and *ΔT_m_* values were determined from the melting curves obtained.

### Isothermal calorimetry (ITC)

Experiments were performed using an iTC200 instrument (MicroCal) at 298 K, with ligands titrated into solutions of SaHPPK using 18×2.2 µL or 13×3.1 µL injections. Data were fitted using Origin software to yield the thermodynamic parameters, Δ*H*, *K*_d_ and N (the binding stoichiometry), assuming a cell volume of 0.2 mL. These were then used to calculate the Gibb's free energy of binding, Δ*G* (−RT.ln*K*_a_), and entropy of binding, Δ*S* (using Δ*G* = Δ*H*−TΔ*S*). For titrations with AMPCPP, SaHPPK and AMPCPP concentrations were typically 30 and 400 µM, respectively. For titrations with ATP, SaHPPK was typically at 70 µM and ATP at 1500 µM. For titrations with HMDP substrate, SaHPPK was typically at 22 µM and HMDP at 500 µM, with AMPCPP added to both solutions at 1 mM where indicated. For titrations with 8-mercaptoguanine, SaHPPK was typically at 11 µM and inhibitor at 300 µM. Inhibitor was prepared as a 200 mM stock solution in DMSO, and diluted into ITC buffer to a nominal concentration of 500 µM, with an equal 0.25% DMSO added to the SaHPPK sample to ensure buffer matching. Experiments were limited by the solubility of 8-mercaptoguanine, estimated to be ∼300 µM from ITC experiments assuming a 1∶1 binding stoichiometry.

### Surface plasmon resonance (SPR)

Minimal biotinylation of SaHPPK was performed as follows: SaHPPK (∼30 nanomolar) in SPR “capture buffer” (50 mM HEPES, pH8.0, 150 mMNaCl, 0.05% (v/v) polysorbate 20, 5 mM DTT, 10 mM MgCl_2_) was incubated with an equimolar concentration of EZ-Link® Sulfo-NHS-LC-LC-Biotin (Pierce) on ice for 2 hours [69]. The biotinylated enzyme was passed through a Superdex 75 (10/300 GL) column equilibrated with SPR capture buffer to remove free biotin. NeutrAvidin (Pierce) was immobilized at 298 K onto a CM5 sensor chip docked in a Biacore T100 instrument (GE Healthcare) as described previously [70], resulting in immobilization levels of approximately 13,000 RU in all flow cells. The biotinylated SaHPPK was bound to the NeutrAvidin chip surface by injecting at 10 µL min^−1^ for 6 min over a single flow cell, typically resulting in immobilization of ∼10,000 RU of SaHPPK. A minimally-biotinylated bovine carbonic anhydrase II protein was captured in a separate flow cell to provide an unrelated negative control. The blank (typically flow cell 1) and captured surfaces were subsequently blocked with three 1 min injections of 1 µg mL^−1^ D-biotin (Sigma) at 10 µL min^−1^.

All SPR binding experiments were performed at 293 K in SPR capture buffer with 5% (v/v) DMSO added. Small molecules (ligands) were serially diluted (either 2- or 3-fold) in SPR binding buffer and injected for 30 sec contact time at 60 µL min^−1^, then allowed to dissociate for 60 sec. SPR running buffer was injected after each ligand injection to ensure complete regeneration of immobilized SaHPPK. Each ligand titration was performed in triplicate. Binding sensorgrams were processed, solvent-corrected and double-referenced using Scrubber software (BioLogic Software, Australia). Responses at equilibrium for each analyte were fitted to a 1∶1 steady-state affinity model available within Scrubber to determine the *K*_d_. Where the dissociation rate was sufficiently slow, binding data were fit globally to a 1∶1 kinetic interaction model that included a mass transport component [71] and the K_d_ determined from the (*k*_d_/*k*_a_) ratio.

### KinaseGlo™ Biochemical assay

The KinaseGlo™ assay kit (Promega) was used to quantify HPPK activity. In this assay, firefly luciferase utilizes the ATP remaining after HPPK catalysis to produce a luminescence signal that is directly proportional to ATP concentration; from this, the HPPK activity can be derived. The enzyme activity and optimum concentration to define kinetic parameters was determined by measuring the initial rate of ATP consumption over a range of HPPK concentrations in the presence and absence of HMDP substrate (data not shown). For kinetic measurements, an optimized HPPK concentration of 7 ng/50 µL assay volume was determined, which allowed for monitoring the first 10% of reactions turnover in a reasonable assay time period (20 min).

Measurements were performed in 96-well plates using assay buffer (100 mMTris-HCl/10 mM MgCl_2_, pH 8.5, 0.01% (w/v) BSA, 0.01% (v/v) Tween 20 and 10 mM β-mercaptoethanol). Typically, 5 µl of test compound (dissolved in 50% DMSO) and 20 µl of enzyme were added to each well followed by 25 µl of assay buffer giving 0.3 µM pterin and 0.2 µM ATP in a total reaction volume 50 µl. After a 20 minute incubation at room temperature, the enzymatic reaction was stopped with 50 µl of KinaseGlo™ reagent. Luminescence was recorded after a further 10 min using the *FLUOstar Optima* plate reader (BMG, Labtech Ltd). Kinetic data and inhibition data were fit to Michaelis-Mentenand sigmoidal dose-response equations respectively, using *GraphPad Prism*.

### Accession Numbers

The coordinates and structure factors of SaHPPK in complex with 8-mercaptoguanine have been deposited in the RCSB Protein Data Bank with accession number 3QBC.

## Supporting Information

Figure S1 A) ITC (left) and SPR data (right) for binding of ATP and AMPCPP to SaHPPK. Equilibrium binding constants (K_d_) are shown. B) SPR data for the binding of ADP and AMP to SaHPPK.
(DOCX)

Figure S2 **Binding of substrate, cofactor and inhibitor to SaHPPK as measured by NMR spectroscopy.** A, Superposition of the ^15^N HSQC spectra of ∼100 µM SaHPPK+10 mM MgSO_4_ with and without 1 mM AMPCPP (left), and the ^15^N HSQC spectra of ∼100 µM SaHPPK+1 mM AMPCPP with and without 1 mM HMDP (right). B, Missing amides are shown on a ribbon representation for apo HPPK (green) and those that additionally disappear in the presence of various saturating ligands (blue). C, Missing amides and CSPs mapped onto surface of HPPK. Residues with missing resonances in the apo enzyme are coloured cyan, whilst those additionally broadened are coloured magenta upon binding of ligands. Residues displaying slow exchange CSPs upon ligand binding in the AMPCPP binary and AMPCPP+8-mercaptoguanine complexes are shaded red, with the hue corresponding to the magnitude of the CSPs. The position of the AMPCPP is modeled from that in the *E. coli* HPPK (1Q0N). D, Missing amides with addition of saturating 8-mercaptoguanine are coloured magenta. CSPs for the slow exchange 8-mercaptoguanine+AMPCPP ternary complex are coloured red. Missing amides are further shown on the ribbon representation in green.
(DOCX)

Figure S3 **^15^N sofast HMQC spectra recorded (20 min per spectrum) over the time period shown for ∼100 µM SaHPPK in complex with either 200 µM HMDP/1 mM AMPCPP (left) and 200 µM oxidized HMDP/1 mM AMPCPP (right).** Several spectral changes are observed in the oxidized HMDP/AMPCPP (right) over time but not in HMDP/AMPCPP (left).
(DOCX)

Figure S4 **^1^H NMR spectra of 5 mM HMDP/5 mM DTT (top) and 20 mM oxidized HMDP (bottom).** Both spectra were recorded in 50 mM Potassium phosphate buffer D_2_O pH 7.9. The very small amount of oxidation of HMDP is just visible at ∼8.6 ppm in the top spectrum. The spectra show the initial purity of the HMDP used in the NMR experiments in S3.
(DOCX)

Figure S5 **CSP data for various ligands binding to SaHPPK.** From top to bottom: HMDP, AMPCPP, Pterin+AMPCPP, 8-mercaptoguanine, 8-mercaptoguanine+AMPCPP. Grey regions indicate residues for which resonances were extensively/fully broadened compared to the apo ^15^N HSQC spectrum, and therefore not observed. The CSPs were calculated using the following equation: Δδ = √(δN^15^×0.154)^2^+(δHN^1^)^2^), δ refers to the chemical shift change in ppm of the resonance relative to the apo SaHPPK ^15^N HSQC spectrum.
(DOCX)

Figure S6 **Comparison of the HYDRONMR calculated ^15^N T1/T2 ratio (600 MHz) for a monomer SaHPPK (black) and the x-ray SaHPPK dimer (red) with the measured ^15^N T1/T2 (blue) for SaHPPK in complex with 8-mercaptoguanine and AMPCPP.**
(DOCX)

Table S1 **^15^N and ^1^HN chemical shifts (ppm) for; SaHPPK, SaHPPK/AMPCPP, SaHPPK/HMDP, SaHPPK/8-mercaptoguanine/AMPCPP, SaHPPK/8-mercaptoguanine.** Chemical shifts in red are from the ^15^Nε1 and ^1^Hε1 resonance of Trp89.
(DOCX)

## References

[1] Fischbach MA, Walsh CT (2009). Antibiotics for emerging pathogens.. Science.

[2] Hidron AI, Low CE, Honig EG, Blumberg HM (2009). Emergence of community-acquired meticillin-resistant Staphylococcus aureus strain USA300 as a cause of necrotising community-onset pneumonia.. Lancet Infect Dis.

[3] David MZ, Daum RS (2010). Community-associated methicillin-resistant Staphylococcus aureus: epidemiology and clinical consequences of an emerging epidemic.. Clin Microbiol Rev.

[4] Naimi TS, LeDell KH, Como-Sabetti K, Borchardt SM, Boxrud DJ (2003). Comparison of community- and health care-associated methicillin-resistant Staphylococcus aureus infection.. JAMA.

[5] Adra M, Lawrence KR (2004). Trimethoprim/sulfamethoxazole for treatment of severe Staphylococcus aureus infections.. Ann Pharmacother.

[6] Proctor RA (2008). Role of folate antagonists in the treatment of methicillin-resistant Staphylococcus aureus infection.. Clin Infect Dis.

[7] Besier S, Zander J, Siegel E, Saum SH, Hunfeld KP (2008). Thymidine-dependent Staphylococcus aureus small-colony variants: human pathogens that are relevant not only in cases of cystic fibrosis lung disease.. J Clin Microbiol.

[8] Zander J, Besier S, Ackermann H, Wichelhaus TA (2010). Synergistic antimicrobial activities of folic acid antagonists and nucleoside analogs.. Antimicrob Agents Chemother.

[9] Frey KM, Liu J, Lombardo MN, Bolstad DB, Wright DL (2009). Crystal structures of wild-type and mutant methicillin-resistant Staphylococcus aureus dihydrofolate reductase reveal an alternate conformation of NADPH that may be linked to trimethoprim resistance.. J Mol Biol.

[10] Black MT, Hodgson J (2005). Novel target sites in bacteria for overcoming antibiotic resistance.. Adv Drug Deliv Rev.

[11] Bermingham A, Derrick JP (2002). The folic acid biosynthesis pathway in bacteria: evaluation of potential for antibacterial drug discovery.. Bioessays.

[12] Swarbrick JD, Iliades P, Simpson JS, Macreadie I (2008). Folate biosynthesis - reappraisal of old and novel targets in the search for new antimicrobials.. The Open Enzyme Inhibition Journal.

[13] Xiao B, Shi G, Chen X, Yan H, Ji X (1999). Crystal structure of 6-hydroxymethyl-7,8-dihydropterin pyrophosphokinase, a potential target for the development of novel antimicrobial agents.. Structure.

[14] Hennig M, Dale GE, D'Arcy A, Danel F, Fischer S (1999). The structure and function of the 6-hydroxymethyl-7,8-dihydropterin pyrophosphokinase from Haemophilus influenzae.. J Mol Biol.

[15] Lawrence MC, Iliades P, Fernley RT, Berglez J, Pilling PA (2005). The three-dimensional structure of the bifunctional 6-hydroxymethyl-7,8-dihydropterin pyrophosphokinase/dihydropteroate synthase of Saccharomyces cerevisiae.. J Mol Biol.

[16] Garcon A, Levy C, Derrick JP (2006). Crystal structure of the bifunctional dihydroneopterin aldolase/6-hydroxymethyl-7,8-dihydropterin pyrophosphokinase from Streptococcus pneumoniae.. J Mol Biol.

[17] Blaszczyk J, Li Y, Cherry S, Alexandratos J, Wu Y (2007). Structure and activity of Yersinia pestis 6-hydroxymethyl-7,8-dihydropterin pyrophosphokinase as a novel target for the development of antiplague therapeutics.. Acta Crystallogr D Biol Crystallogr.

[18] Pemble CW, Mehta PK, Mehra S, Li Z, Nourse A (2010). Crystal structure of the 6-hydroxymethyl-7,8-dihydropterin pyrophosphokinase•dihydropteroate synthase bifunctional enzyme from Francisella tularensis.. PLoS One.

[19] Blaszczyk J, Shi G, Li Y, Yan H, Ji X (2004). Reaction trajectory of pyrophosphoryl transfer catalyzed by 6-hydroxymethyl-7,8-dihydropterin pyrophosphokinase.. Structure.

[20] Blaszczyk J, Li Y, Wu Y, Shi G, Ji X (2004). Essential roles of a dynamic loop in the catalysis of 6-hydroxymethyl-7,8-dihydropterin pyrophosphokinase.. Biochemistry.

[21] Blaszczyk J, Shi G, Yan H, Ji X (2000). Catalytic center assembly of HPPK as revealed by the crystal structure of a ternary complex at 1.25 A resolution.. Structure.

[22] Blaszczyk J, Li Y, Shi G, Yan H, Ji X (2003). Dynamic roles of arginine residues 82 and 92 of Escherichia coli 6-hydroxymethyl-7,8-dihydropterin pyrophosphokinase: crystallographic studies.. Biochemistry.

[23] Bermingham A, Bottomley JR, Primrose WU, Derrick JP (2000). Equilibrium and kinetic studies of substrate binding to 6-hydroxymethyl-7,8-dihydropterin pyrophosphokinase from Escherichia coli.. J Biol Chem.

[24] Li Y, Gong Y, Shi G, Blaszczyk J, Ji X (2002). Chemical transformation is not rate-limiting in the reaction catalyzed by Escherichia coli 6-hydroxymethyl-7,8-dihydropterin pyrophosphokinase.. Biochemistry.

[25] Shi G, Gong Y, Savchenko A, Zeikus JG, Xiao B (2000). Dissecting the nucleotide binding properties of Escherichia coli 6-hydroxymethyl-7,8-dihydropterin pyrophosphokinase with fluorescent 3′(2)′-o-anthraniloyladenosine 5′-triphosphate.. Biochim Biophys Acta.

[26] Stammers DK, Achari A, Somers DO, Bryant PK, Rosemond J (1999). 2.0 A X-ray structure of the ternary complex of 7,8-dihydro-6-hydroxymethylpterinpyrophosphokinase from Escherichia coli with ATP and a substrate analogue.. FEBS Lett.

[27] Shi G, Blaszczyk J, Ji X, Yan H (2001). Bisubstrate analogue inhibitors of 6-hydroxymethyl-7,8-dihydropterin pyrophosphokinase: synthesis and biochemical and crystallographic studies.. J Med Chem.

[28] Pantoliano MW, Petrella EC, Kwasnoski JD, Lobanov VS, Myslik J (2001). High-density miniaturized thermal shift assays as a general strategy for drug discovery.. Journal of Biomolecular Screening.

[29] Bagby S, Tong KI, Liu DJ, Alattia JR, Ikura M (1997). The button test: A small scale method using microdialysis cells for assessing protein solubility at concentrations suitable for NMR.. Journal of Biomolecular Nmr.

[30] Rush TS, Grant JA, Mosyak L, Nicholls A (2005). A shape-based 3-D scaffold hopping method and its application to a bacterial protein-protein interaction.. Journal of Medicinal Chemistry.

[31] Lo MC, Aulabaugh A, Jin GX, Cowling R, Bard J (2004). Evaluation of fluorescence-based thermal shift assays for hit identification in drug discovery.. Analytical Biochemistry.

[32] Niesen FH, Berglund H, Vedadi M (2007). The use of differential scanning fluorimetry to detect ligand interactions that promote protein stability.. Nat Protoc.

[33] Talarico TL, Dev IK, Dallas WS, Ferone R, Ray PH (1991). Purification and partial characterization of 7,8-dihydro-6-hydroxymethylpterin-pyrophosphokinase and 7,8-dihydropteroate synthase from Escherichia coli MC4100.. Journal of bacteriology.

[34] Huber W (2005). A new strategy for improved secondary screening and lead optimization using high-resolution SPR characterization of compound-target interactions.. J Mol Recognit.

[35] Chhabra S, Newman J, Peat TS, Fernley RT, Caine J (2010). Crystallization and preliminary X-ray analysis of 6-hydroxymethyl-7,8-dihydropterin pyrophosphokinase from Staphylococcus aureus.. Acta Crystallogr Sect F Struct Biol Cryst Commun.

[36] Krissinel E, Henrick K (2004). Secondary-structure matching (SSM), a new tool for fast protein structure alignment in three dimensions.. Acta Crystallogr D Biol Crystallogr.

[37] Lescop E, Lu ZW, Liu Q, Xu HM, Li GY (2009). Dynamics of the Conformational Transitions in the Assembling of the Michaelis Complex of a Bisubstrate Enzyme: A N-15 Relaxation Study of Escherichia coli 6-Hydroxymethyl-7,8-dihydropterin Pyrophosphokinase.. Biochemistry.

[38] Garcia de la Torre J, Huertas ML, Carrasco B (2000). HYDRONMR: prediction of NMR relaxation of globular proteins from atomic-level structures and hydrodynamic calculations.. J Magn Reson.

[39] Ruckert M, Otting G (2000). Alignment of biological macromolecules in novel nonionic liquid crystalline media for NMR experiments.. Journal of the American Chemical Society.

[40] Zweckstetter M, Bax A (2000). Prediction of sterically induced alignment in a dilute liquid crystalline phase: Aid to protein structure determination by NMR.. Journal of the American Chemical Society.

[41] Derrick JP (2008). The structure and mechanism of 6-hydroxymethyl-7,8-dihydropterin pyrophosphokinase.. Vitam Horm.

[42] Beavo JA, Rogers NL, Crofford OB, Hardman JG, Sutherland EW (1970). Effects of xanthine derivatives on lipolysis and on adenosine 3′,5′-monophosphate phosphodiesterase activity.. Molecular pharmacology.

[43] Kalra S, Jena G, Tikoo K, Mukhopadhyay AK (2007). Preferential inhibition of xanthine oxidase by 2-amino-6-hydroxy-8-mercaptopurine and 2-amino-6-purine thiol.. BMC biochemistry.

[44] Michael MA, Cottam HB, Smee DF, Robins RK, Kini GD (1993). Alkylpurines as immunopotentiating agents. Synthesis and antiviral activity of certain alkylguanines.. Journal of medicinal chemistry.

[45] Miller RL, Ramsey GA, Krenitsky TA, Elion GB (1972). Guanine phosphoribosyltransferase from Escherichia coli, specificity and properties.. Biochemistry.

[46] Stoeckler JD, Cambor C, Kuhns V, Chu SH, Parks RE (1982). Inhibitors of purine nucleoside phosphorylase, C(8) and C(5′) substitutions.. Biochemical pharmacology.

[47] Goodman MG, Weigle WO (1983). Activation of lymphocytes by a thiol-derivatized nucleoside: characterization of cellular parameters and responsive subpopulations.. Journal of immunology.

[48] Hevener KE, Yun MK, Qi JJ, Kerr ID, Babaoglu K (2010). Structural Studies of Pterin-Based Inhibitors of Dihydropteroate Synthase.. Journal of Medicinal Chemistry.

[49] Gibson CL, Huggan JK, Kennedy A, Kiefer L, Lee JH (2009). Diversity oriented syntheses of fused pyrimidines designed as potential antifolates.. Organic & biomolecular chemistry.

[50] Swarbrick JD, Buyya S, Gunawardana D, Gayler KR, McLennan AG (2005). Structure and substrate-binding mechanism of human Ap4A hydrolase.. J Biol Chem.

[51] Li G, Felczak K, Shi G, Yan H (2006). Mechanism of the conformational transitions in 6-hydroxymethyl-7,8-dihydropterin pyrophosphokinase as revealed by NMR spectroscopy.. Biochemistry.

[52] Bax A (2003). Weak alignment offers new NMR opportunities to study protein structure and dynamics.. Protein Sci.

[53] Lipsitz RS, Tjandra N (2004). Residual dipolar couplings in NMR structure analysis.. Annu Rev Biophys Biomol Struct.

[54] Vajpai N, Strauss A, Fendrich G, Cowan-Jacob SW, Manley PW (2008). Solution conformations and dynamics of ABL kinase-inhibitor complexes determined by NMR substantiate the different binding modes of imatinib/nilotinib and dasatinib.. The Journal of biological chemistry.

[55] Storoni LC, McCoy AJ, Read RJ (2004). Likelihood-enhanced fast rotation functions.. Acta Crystallogr D Biol Crystallogr.

[56] Murshudov GN, Vagin AA, Dodson EJ (1997). Refinement of macromolecular structures by the maximum-likelihood method.. Acta Crystallographica Section D-Biological Crystallography.

[57] Emsley P, Cowtan K (2004). Coot: model-building tools for molecular graphics.. Acta Crystallographica Section D-Biological Crystallography.

[58] Laskowski RA, MacArthur MW, Moss DS, Thornton JM (1993). PROCHECK: a program to check the stereochemical quality of protein structures.. J Appl Cryst.

[59] Talluri S, Wagner G (1996). An optimized 3D NOESY-HSQC.. J Magn Reson B.

[60] Schanda P, Kupce E, Brutscher B (2005). SOFAST-HMQC experiments for recording two-dimensional heteronuclear correlation spectra of proteins within a few seconds.. J Biomol NMR.

[61] Zhu G, Xia YL, Nicholson LK, Sze KH (2000). Protein dynamics measurements by TROSY-based NMR experiments.. Journal of Magnetic Resonance.

[62] Farrow NA, Muhandiram R, Singer AU, Pascal SM, Kay CM (1994). Backbone Dynamics of a Free and a Phosphopeptide-Complexed Src Homology-2 Domain Studied by N-15 Nmr Relaxation.. Biochemistry.

[63] Delaglio F, Grzesiek S, Vuister GW, Zhu G, Pfeifer J (1995). NMRPipe: a multidimensional spectral processing system based on UNIX pipes.. J Biomol NMR.

[64] Bartels C, Xia TH, Billeter M, Guntert P, Wuthrich K (1995). The Program Xeasy for Computer-Supported Nmr Spectral-Analysis of Biological Macromolecules.. Journal of Biomolecular Nmr.

[65] Goddard TD, Kneller D (2001). SPARKY 3 ed.

[66] Ottiger M, Delaglio F, Bax A (1998). Measurement of J and dipolar couplings from simplified two-dimensional NMR spectra.. J Magn Reson.

[67] LigPrep (2011). Version 2.3.

[68] ROCS (2005). Version 2.1.1.

[69] Papalia G, Myszka D (2010). Exploring minimal biotinylation conditions for biosensor analysis using capture chips.. Analytical Biochemistry.

[70] Katsamba P, Carroll K, Ahlsen G, Bahna F, Vendome J (2009). Linking molecular affinity and cellular specificity in cadherin-mediated adhesion.. Proc Natl Acad Sci U S A.

[71] Myszka DG, Morton TA, Doyle ML, Chaiken IM (1997). Kinetic analysis of a protein antigen-antibody interaction limited by mass transport on an optical biosensor.. Biophys Chem.

