# Supplementary figures and images for: Structure of S. aureus HPPK and the Discovery of a New Substrate Site Inhibitor
Source: PLoS One. 2012 Jan 19;7(1):e29444. doi: 10.1371/journal.pone.0029444 (PMC3261883; doi:10.1371/journal.pone.0029444)

**A**


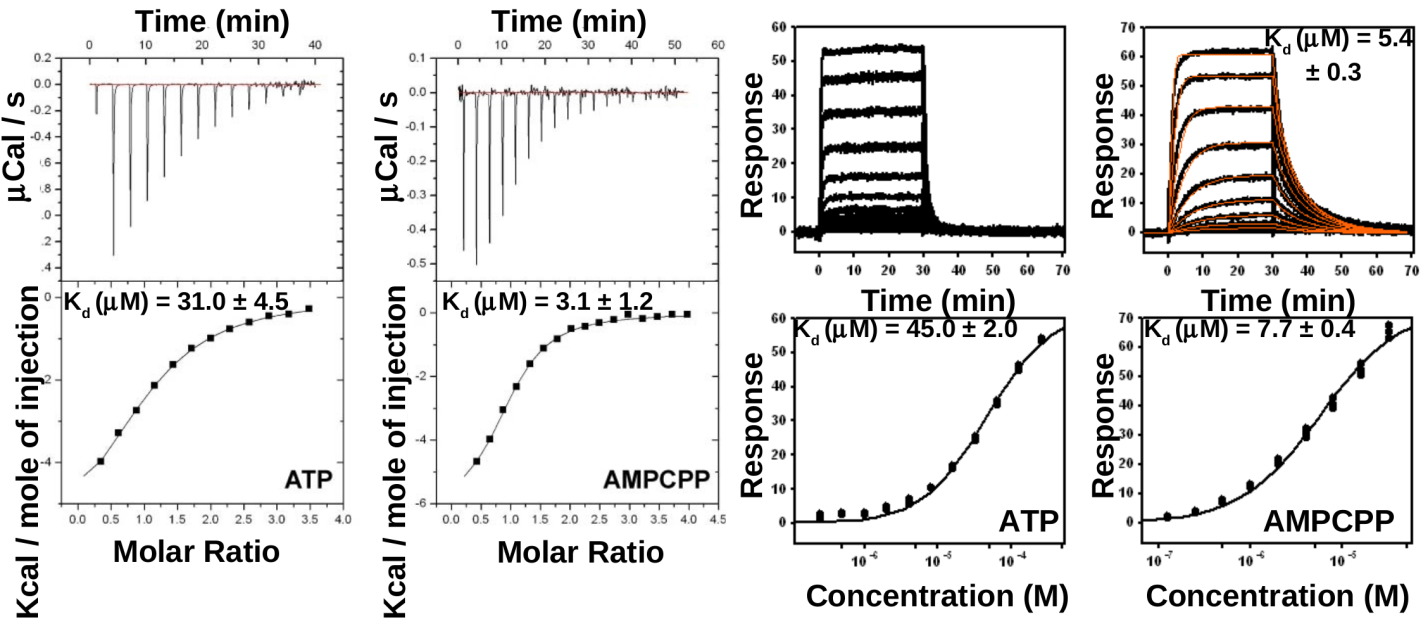


**B**


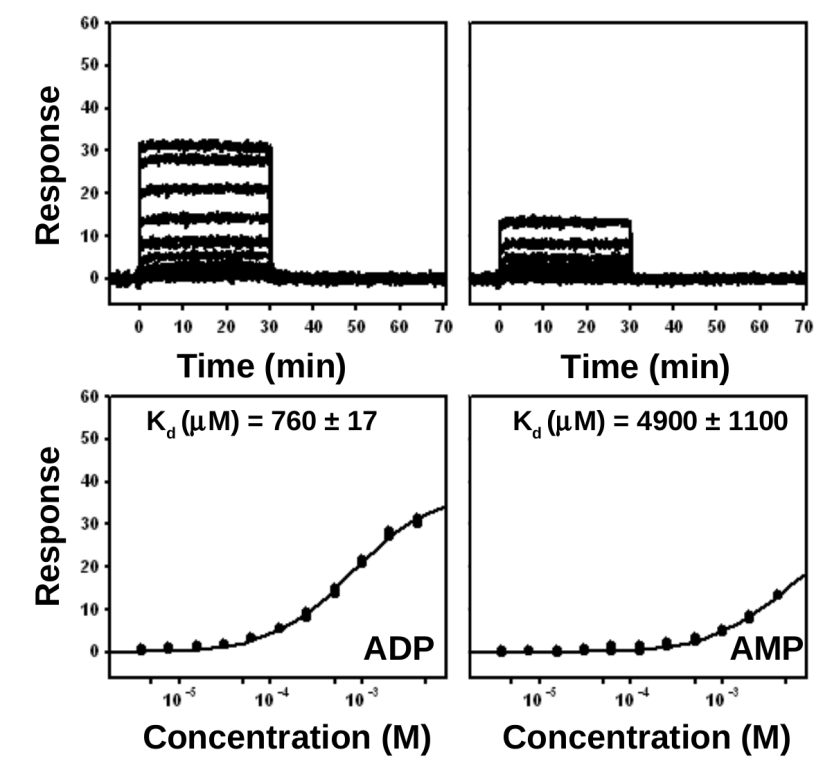

Supplement: Figure S1 — A) ITC (left) and SPR data (right) for binding of ATP and AMPCPP to SaHPPK. Equilibrium binding constants (Kd) are shown. B) SPR data for the binding of ADP and AMP to SaHPPK. (DOCX) [file pone.0029444.s001.docx]

**
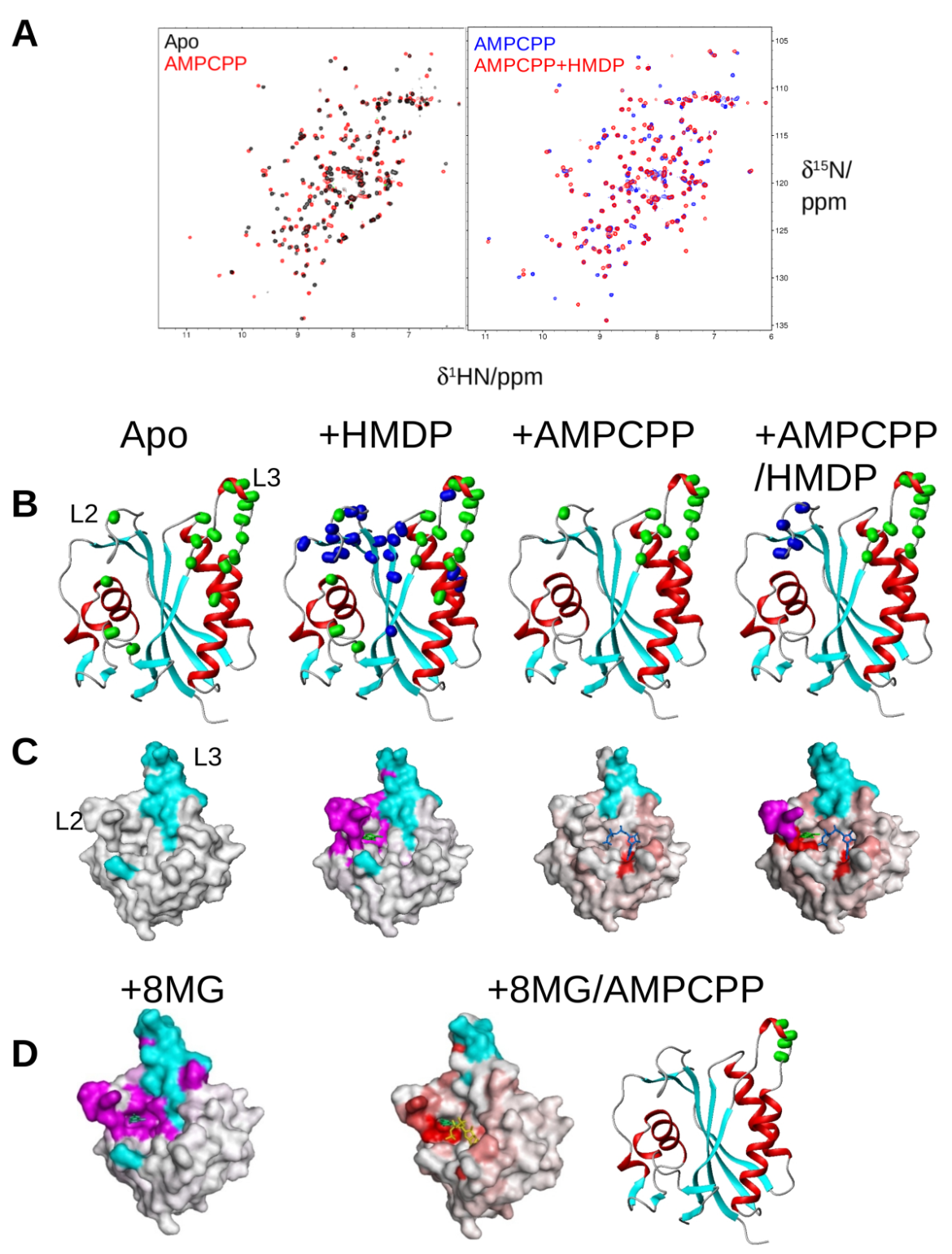
**

Supplement: Figure S2 — Binding of substrate, cofactor and inhibitor to SaHPPK as measured by NMR spectroscopy. A, Superposition of the 15N HSQC spectra of ∼100 µM SaHPPK+10 mM MgSO4 with and without 1 mM AMPCPP (left), and the 15N HSQC spectra of ∼100 µM SaHPPK+1 mM AMPCPP with and without 1 mM HMDP (right). B, Missing amides are shown on a ribbon representation for apo HPPK (green) and those that additionally disappear in the presence of various saturating ligands (blue). C, Missing amides and CSPs mapped onto surface of HPPK. Residues with missing resonances in the apo enzyme are coloured cyan, whilst those additionally broadened are coloured magenta upon binding of ligands. Residues displaying slow exchange CSPs upon ligand binding in the AMPCPP binary and AMPCPP+8-mercaptoguanine complexes are shaded red, with the hue corresponding to the magnitude of the CSPs. The position of the AMPCPP is modeled from that in the E. coli HPPK (1Q0N). D, Missing amides with addition of saturating 8-mercaptoguanine are coloured magenta. CSPs for the slow exchange 8-mercaptoguanine+AMPCPP ternary complex are coloured red. Missing amides are further shown on the ribbon representation in green. (DOCX) [file pone.0029444.s002.docx]

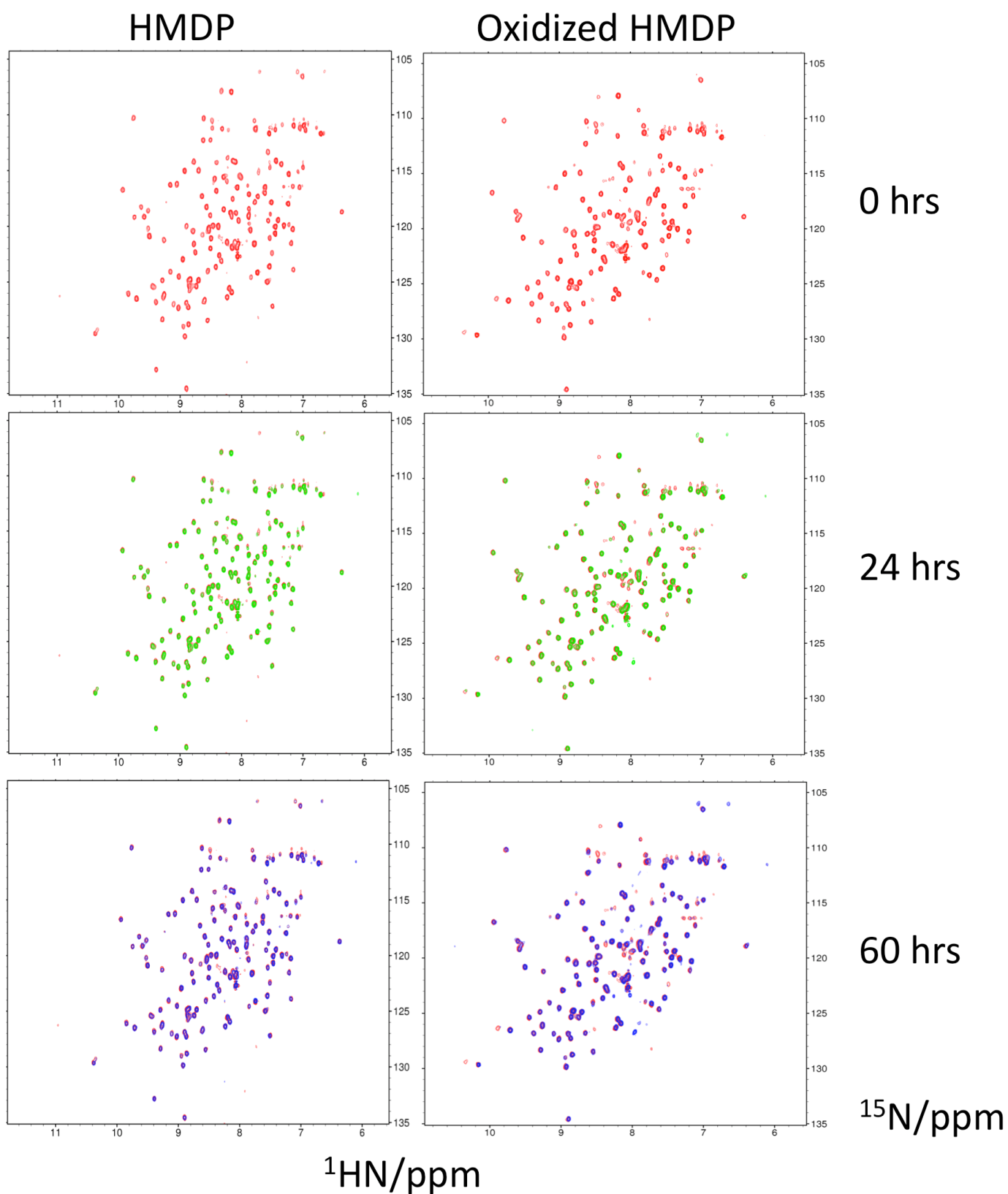

Supplement: Figure S3 — 15N sofast HMQC spectra recorded (20 min per spectrum) over the time period shown for ∼100 µM SaHPPK in complex with either 200 µM HMDP/1 mM AMPCPP (left) and 200 µM oxidized HMDP/1 mM AMPCPP (right). Several spectral changes are observed in the oxidized HMDP/AMPCPP (right) over time but not in HMDP/AMPCPP (left). (DOCX) [file pone.0029444.s003.docx]

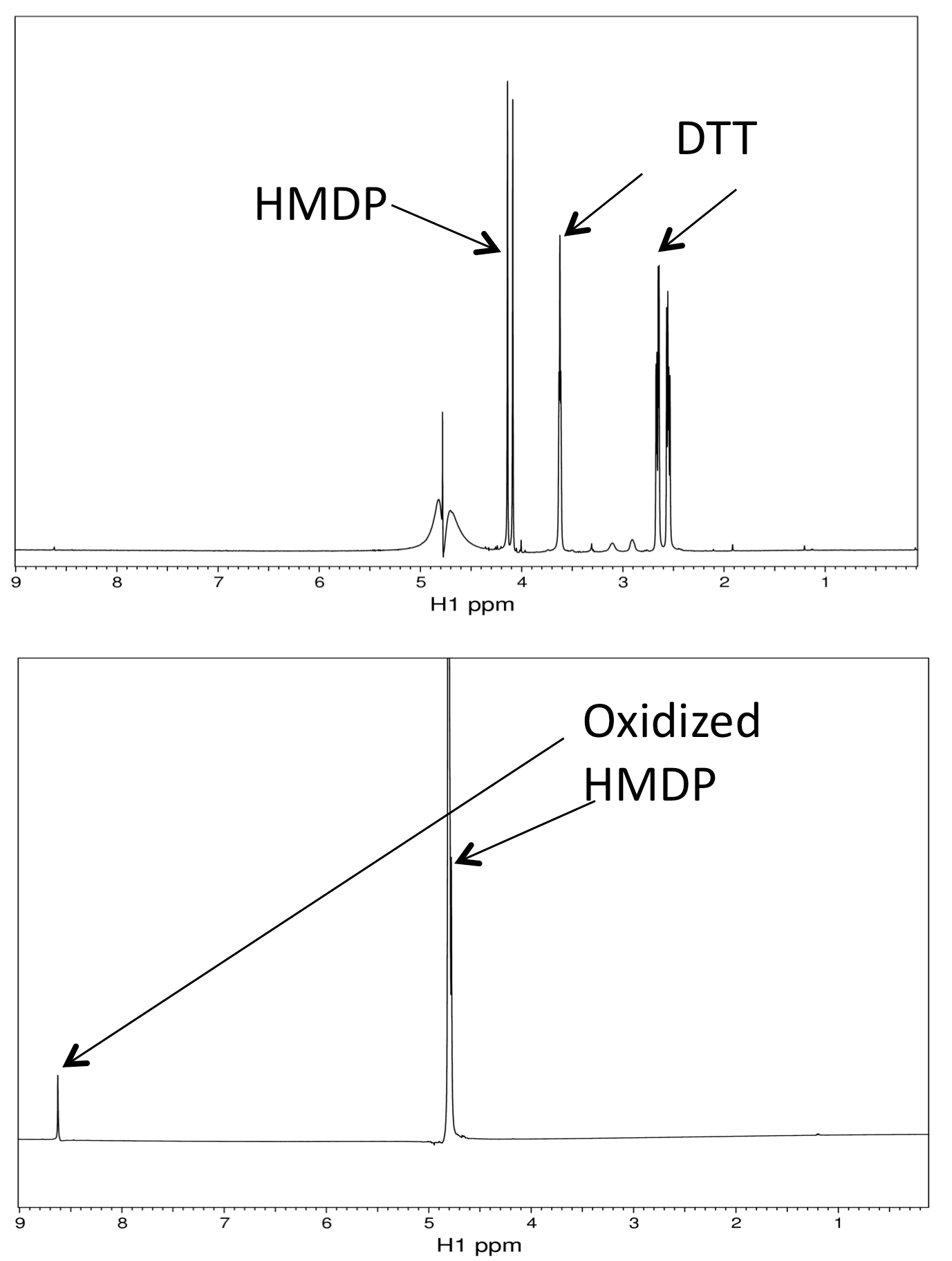


HMP

Supplement: Figure S4 — 1H NMR spectra of 5 mM HMDP/5 mM DTT (top) and 20 mM oxidized HMDP (bottom). Both spectra were recorded in 50 mM Potassium phosphate buffer D2O pH 7.9. The very small amount of oxidation of HMDP is just visible at ∼8.6 ppm in the top spectrum. The spectra show the initial purity of the HMDP used in the NMR experiments in S3. (DOCX) [file pone.0029444.s004.docx]

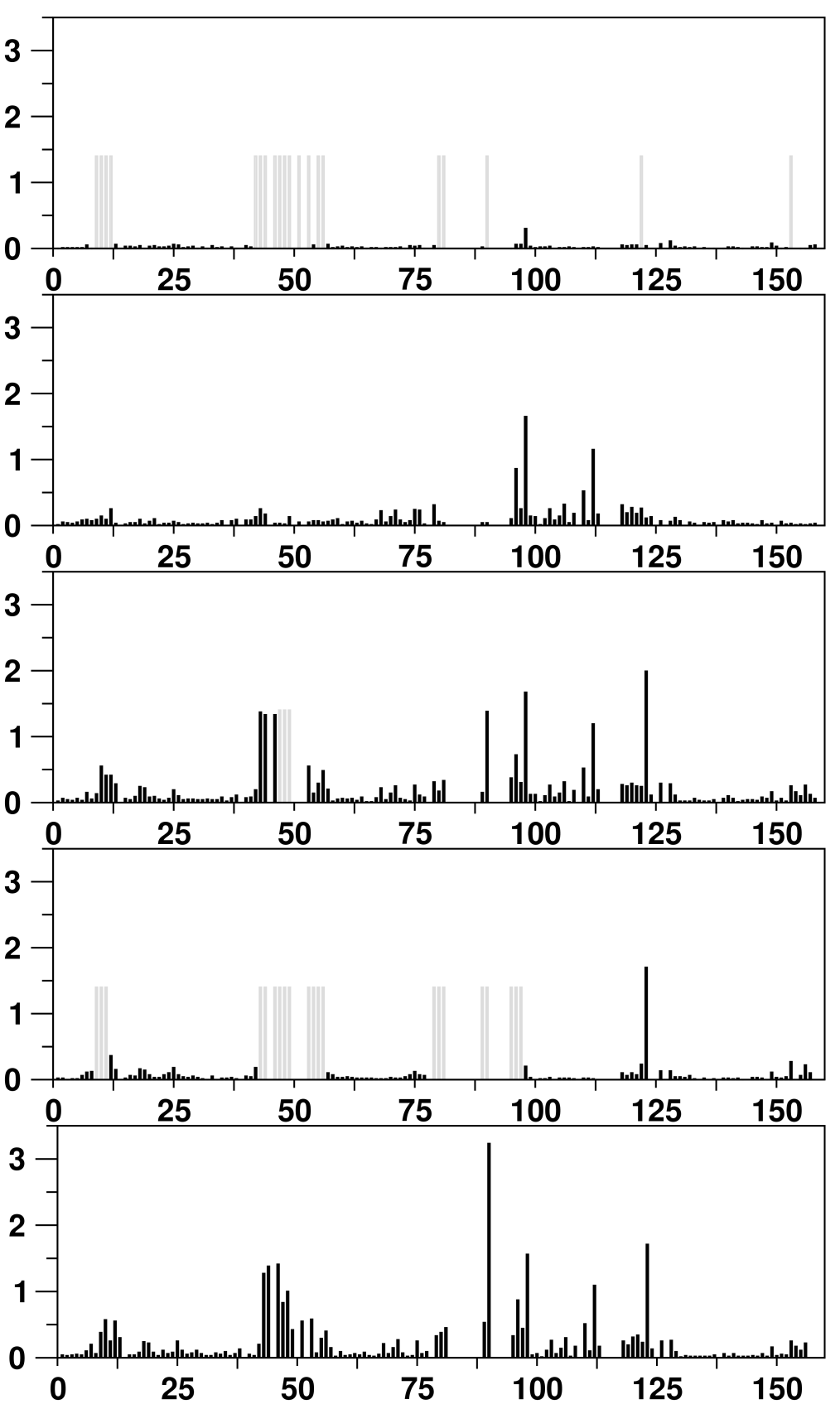

Supplement: Figure S5 — CSP data for various ligands binding to SaHPPK. From top to bottom: HMDP, AMPCPP, Pterin+AMPCPP, 8-mercaptoguanine, 8-mercaptoguanine+AMPCPP. Grey regions indicate residues for which resonances were extensively/fully broadened compared to the apo 15N HSQC spectrum, and therefore not observed. The CSPs were calculated using the following equation: Δδ = √(δN15×0.154)2+(δHN1)2), δ refers to the chemical shift change in ppm of the resonance relative to the apo SaHPPK 15N HSQC spectrum. (DOCX) [file pone.0029444.s005.docx]

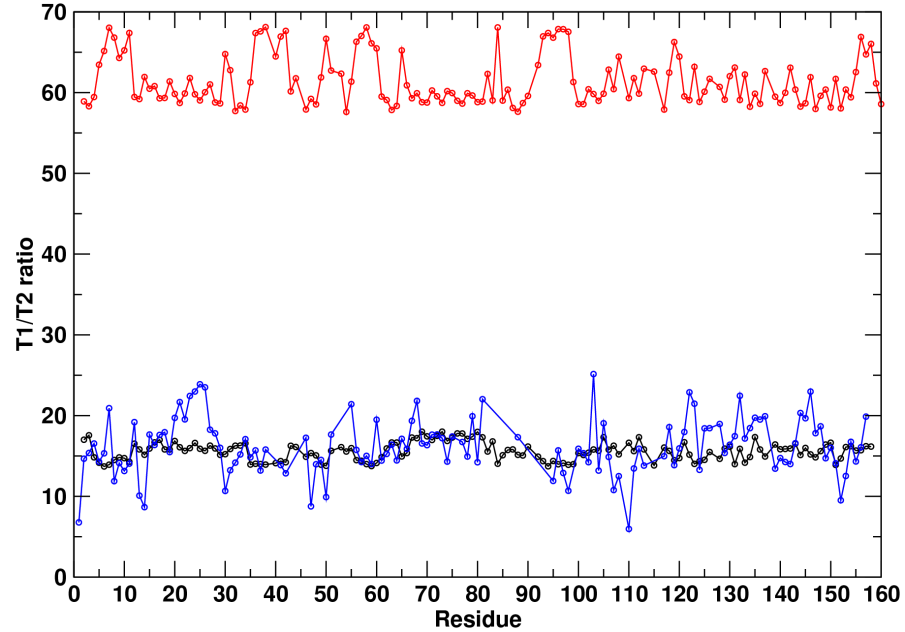

Supplement: Figure S6 — Comparison of the HYDRONMR calculated 15N T1/T2 ratio (600 MHz) for a monomer SaHPPK (black) and the x-ray SaHPPK dimer (red) with the measured 15N T1/T2 (blue) for SaHPPK in complex with 8-mercaptoguanine and AMPCPP. (DOCX) [file pone.0029444.s006.docx]
