# Supplementary material for: Structure of S. aureus HPPK and the Discovery of a New Substrate Site Inhibitor
Source: PLoS One. 2012 Jan 19;7(1):e29444. doi: 10.1371/journal.pone.0029444 (PMC3261883; doi:10.1371/journal.pone.0029444)
Supplement: Table S1 — 15N and 1HN chemical shifts (ppm) for; SaHPPK, SaHPPK/AMPCPP, SaHPPK/HMDP, SaHPPK/8-mercaptoguanine/AMPCPP, SaHPPK/8-mercaptoguanine. Chemical shifts in red are from the 15Nε1 and 1Hε1 resonance of Trp89. (DOCX) [file pone.0029444.s007.docx]

| Res. No | Res. type | apo | apo | ampcpp | ampcpp | HMDP/ampcpp | HMDP/ampcpp | 8MG/ampcpp | 8MG/ampcpp | 8MG | 8MG |
| --- | --- | --- | --- | --- | --- | --- | --- | --- | --- | --- | --- |
|  |  | **^15^N** | **^1^HN** | **^15^N** | **^1^HN** | **^15^N** | **^1^HN** | **^15^N** | **^1^HN** | **^15^N** | **^1^HN** |
| 1 | **MET** | 121.66 | 7.97 | 121.74 | 8.00 | 121.75 | 8.00 | 121.73 | 8.00 | 121.52 | 7.97 |
| 2 | **ILE** | 126.77 | 9.00 | 127.19 | 9.00 | 127.25 | 9.01 | 127.13 | 8.99 | 126.71 | 9.01 |
| 3 | **GLN** | 126.18 | 8.25 | 126.28 | 8.22 | 126.34 | 8.23 | 126.23 | 8.21 | 126.26 | 8.26 |
| 4 | **ALA** | 129.84 | 8.89 | 129.77 | 8.90 | 129.85 | 8.91 | 129.76 | 8.90 | 129.95 | 8.87 |
| 5 | **TYR** | 115.28 | 8.65 | 115.00 | 8.68 | 114.96 | 8.69 | 114.98 | 8.66 | 115.33 | 8.66 |
| 6 | **LEU** | 121.38 | 9.26 | 121.24 | 9.20 | 121.24 | 9.24 | 121.03 | 9.16 | 121.58 | 9.31 |
| 7 | **GLY** | 109.85 | 9.62 | 109.68 | 9.70 | 110.29 | 9.76 | 110.55 | 9.78 | 110.58 | 9.59 |
| 8 | **LEU** | 125.26 | 8.89 | 125.63 | 8.84 | 125.44 | 8.84 | 125.16 | 8.77 | 124.62 | 8.76 |
| 9 | **GLY** | 107.11 | 8.39 | 106.77 | 8.32 | 86.86 | 8.33 | 107.33 | 8.01 |  |  |
| 10 | **SER** | 114.01 | 8.57 | 115.12 | 8.55 | 111.58 | 8.20 | 112.28 | 8.06 |  |  |
| 11 | **ASN** | 125.77 | 8.46 | 126.25 | 8.48 | 125.56 | 8.83 | 124.19 | 8.53 |  |  |
| 12 | **ILE** | 118.63 | 7.30 | 117.66 | 7.11 | 116.62 | 7.06 | 116.18 | 6.91 | 116.87 | 7.07 |
| 13 | **GLY** | 111.87 | 8.20 | 111.85 | 8.23 | 111.18 | 8.47 | 111.10 | 8.47 | 111.07 | 8.24 |
| 14 | **ASP** |  |  | 122.63 | 8.18 | 121.58 | 8.09 | 121.10 | 8.14 |  |  |
| 15 | **ARG** | 124.60 | 7.83 | 124.62 | 7.83 | 124.38 | 7.82 | 124.98 | 7.84 | 124.73 | 7.81 |
| 16 | **GLU** | 117.37 | 8.66 | 117.26 | 8.64 | 117.27 | 8.64 | 117.27 | 8.63 | 117.16 | 8.62 |
| 17 | **SER** | 114.41 | 7.51 | 114.33 | 7.46 | 114.10 | 7.42 | 113.97 | 7.41 | 114.23 | 7.44 |
| 18 | **GLN** | 119.39 | 7.93 | 119.08 | 7.98 | 119.09 | 8.15 | 119.05 | 8.15 | 119.33 | 8.07 |
| 19 | **LEU** | 117.02 | 7.59 | 116.89 | 7.60 | 117.79 | 7.77 | 117.70 | 7.77 | 117.47 | 7.70 |
| 20 | **ASN** | 116.02 | 8.02 | 116.29 | 8.06 | 116.43 | 8.06 | 116.45 | 8.02 | 116.35 | 8.06 |
| 21 | **ASP** | 122.63 | 8.87 | 122.91 | 8.97 | 122.95 | 8.95 | 122.81 | 8.90 | 122.78 | 8.90 |
| 22 | **ALA** | 122.11 | 8.11 | 122.09 | 8.10 | 121.95 | 8.14 | 121.74 | 8.18 | 122.03 | 8.13 |
| 23 | **ILE** | 117.40 | 7.72 | 117.54 | 7.69 | 117.33 | 7.72 | 117.16 | 7.73 | 117.31 | 7.77 |
| 24 | **LYS** | 119.09 | 7.40 | 119.24 | 7.38 | 119.41 | 7.39 | 119.48 | 7.38 | 119.43 | 7.46 |
| 25 | **ILE** | 119.78 | 7.88 | 119.28 | 7.89 | 119.51 | 8.04 | 119.49 | 8.07 | 119.54 | 8.03 |
| 26 | **LEU** | 120.29 | 8.42 | 120.44 | 8.46 | 120.27 | 8.52 | 120.16 | 8.51 | 120.26 | 8.48 |
| 27 | **ASN** | 111.43 | 7.83 | 111.43 | 7.84 | 111.34 | 7.79 | 111.20 | 7.76 | 111.27 | 7.78 |
| 28 | **GLU** | 116.34 | 7.58 | 116.39 | 7.58 | 116.45 | 7.60 | 116.47 | 7.60 | 116.34 | 7.58 |
| 29 | **TYR** | 120.08 | 7.34 | 119.96 | 7.33 | 119.88 | 7.31 | 119.70 | 7.26 | 119.89 | 7.31 |
| 30 | **ASN** | 124.67 | 8.79 | 124.74 | 8.82 | 124.77 | 8.84 | 124.80 | 8.85 | 124.70 | 8.83 |
| 31 | **GLY** | 110.12 | 8.59 | 110.24 | 8.60 | 110.25 | 8.61 | 110.30 | 8.60 | 110.00 | 8.60 |
| 32 | **ILE** | 121.37 | 7.70 | 121.50 | 7.74 | 121.47 | 7.74 | 121.42 | 7.73 | 121.42 | 7.69 |
| 33 | **SER** | 118.89 | 8.08 | 118.85 | 8.07 | 118.89 | 8.10 | 118.98 | 8.14 | 118.94 | 8.11 |
| 34 | **VAL** | 127.17 | 8.80 | 127.25 | 8.83 | 127.23 | 8.84 | 127.16 | 8.84 | 127.15 | 8.80 |
| 35 | **SER** | 123.86 | 9.21 | 123.95 | 9.16 | 124.06 | 9.15 | 124.01 | 9.13 | 123.81 | 9.19 |
| 36 | **ASN** | 119.09 | 7.87 |  |  | 119.16 | 7.88 | 119.10 | 7.87 | 119.03 | 7.87 |
| 37 | **ILE** | 122.32 | 8.30 | 122.61 | 8.36 | 122.61 | 8.35 | 122.54 | 8.32 | 122.51 | 8.30 |
| 38 | **SER** | 124.79 | 9.10 | 124.67 | 9.03 | 124.54 | 9.02 | 124.41 | 9.00 | 124.67 | 9.10 |
| 39 | **PRO** |  |  |  |  |  |  |  |  |  |  |
| 40 | **ILE** | 119.71 | 8.40 | 119.94 | 8.46 | 119.94 | 8.46 | 119.91 | 8.43 | 119.79 | 8.44 |
| 41 | **TYR** | 126.62 | 9.27 | 126.12 | 9.23 | 126.15 | 9.24 | 126.43 | 9.27 | 126.32 | 9.24 |
| 42 | **GLU** | 124.80 | 9.88 | 125.63 | 9.93 | 126.01 | 9.84 | 125.96 | 9.82 | 125.94 | 9.83 |
| 43 | **THR** | 124.59 | 8.62 | 124.65 | 8.39 | 119.19 | 9.74 | 118.63 | 9.51 |  |  |
| 44 | **ALA** | 130.98 | 8.68 | 131.82 | 8.80 | 127.16 | 7.49 | 126.88 | 7.40 |  |  |
| 45 | **PRO** |  |  |  |  |  |  |  |  |  |  |
| 46 | **VAL** | 120.82 | 8.32 | 120.77 | 8.36 | 125.68 | 9.43 | 124.98 | 9.57 |  |  |
| 47 | **GLY** | 112.84 | 8.43 | 112.86 | 8.41 |  |  | 116.40 | 9.06 |  |  |
| 48 | **TYR** | 120.73 | 8.15 | 120.84 | 8.17 |  |  | 127.23 | 8.01 |  |  |
| 49 | **THR** | 116.54 | 7.87 | 117.40 | 7.84 |  |  | 116.24 | 8.28 |  |  |
| 50 | **GLU** |  |  | 121.88 | 8.39 | 123.74 | 7.16 | 124.30 | 6.98 |  |  |
| 51 | **GLN** | 120.14 | 7.87 | 119.82 | 7.86 |  |  | 116.58 | 7.78 |  |  |
| 52 | **PRO** |  |  |  |  |  |  |  |  |  |  |
| 53 | **ASN** | 118.90 | 8.45 | 119.11 | 8.50 | 115.54 | 8.28 | 115.42 | 8.24 | 115.49 | 8.21 |
| 54 | **PHE** | 121.58 | 8.68 | 121.50 | 8.76 | 120.74 | 8.63 | 121.07 | 8.69 | 120.90 | 8.70 |
| 55 | **LEU** | 115.49 | 8.17 | 114.94 | 8.14 | 114.30 | 8.41 | 113.49 | 8.17 |  |  |
| 56 | **ASN** | 117.66 | 9.22 | 117.68 | 9.18 | 120.16 | 9.51 | 119.77 | 9.44 | 119.95 | 9.47 |
| 57 | **LEU** | 114.83 | 8.95 | 114.40 | 8.93 | 114.17 | 8.76 | 113.86 | 8.87 | 114.72 | 8.84 |
| 58 | **CYS** | 118.40 | 9.51 | 118.75 | 9.58 | 118.58 | 9.51 | 118.41 | 9.47 | 118.10 | 9.45 |
| 59 | **VAL** | 116.28 | 9.09 | 115.81 | 9.02 | 116.24 | 9.04 | 115.72 | 9.03 | 116.36 | 9.10 |
| 60 | **GLU** | 126.68 | 8.84 | 126.67 | 8.85 | 126.83 | 8.88 | 126.48 | 8.84 | 126.75 | 8.86 |
| 61 | **ILE** | 119.77 | 8.91 | 120.03 | 8.89 | 119.99 | 8.89 | 119.81 | 8.87 | 119.47 | 8.89 |
| 62 | **GLN** | 120.20 | 8.74 | 120.47 | 8.77 | 120.41 | 8.78 | 120.39 | 8.76 | 119.99 | 8.73 |
| 63 | **THR** | 119.17 | 9.60 | 119.18 | 9.58 | 119.19 | 9.58 | 119.12 | 9.58 | 119.06 | 9.60 |
| 64 | **THR** | 115.43 | 7.93 | 115.54 | 7.97 | 115.53 | 8.00 | 115.53 | 7.97 | 115.42 | 7.93 |
| 65 | **LEU** | 124.19 | 8.54 | 123.99 | 8.53 | 124.01 | 8.53 | 123.89 | 8.52 | 124.07 | 8.51 |
| 66 | **THR** | 112.24 | 8.60 | 112.26 | 8.61 | 112.24 | 8.61 | 112.16 | 8.59 | 112.19 | 8.60 |
| 67 | **VAL** | 121.84 | 8.77 | 122.36 | 8.75 | 122.33 | 8.75 | 122.14 | 8.74 | 121.82 | 8.77 |
| 68 | **LEU** | 118.94 | 7.24 | 120.21 | 7.14 | 120.22 | 7.15 | 120.07 | 7.15 | 118.90 | 7.24 |
| 69 | **GLN** | 120.99 | 7.57 | 121.09 | 7.52 | 121.08 | 7.53 | 121.05 | 7.51 | 121.00 | 7.55 |
| 70 | **LEU** | 122.26 | 8.83 | 121.55 | 8.79 | 121.56 | 8.77 | 121.53 | 8.74 | 122.14 | 8.81 |
| 71 | **LEU** | 122.08 | 8.44 | 121.44 | 8.25 | 121.38 | 8.23 | 121.25 | 8.20 | 122.09 | 8.42 |
| 72 | **GLU** | 117.98 | 7.57 | 117.92 | 7.48 | 117.84 | 7.50 | 117.68 | 7.48 | 117.89 | 7.55 |
| 73 | **CYS** | 116.91 | 7.58 | 116.76 | 7.61 | 116.94 | 7.61 | 116.99 | 7.57 | 117.08 | 7.54 |
| 74 | **CYS** | 123.06 | 8.36 | 122.68 | 8.34 | 123.05 | 8.33 | 123.10 | 8.30 | 123.53 | 8.30 |
| 75 | **LEU** | 121.06 | 8.57 | 119.80 | 8.43 | 119.90 | 8.39 | 120.07 | 8.36 | 120.88 | 8.45 |
| 76 | **LYS** | 119.44 | 8.43 | 120.61 | 8.63 | 120.10 | 8.53 | 119.83 | 8.44 | 119.18 | 8.41 |
| 77 | **THR** | 118.93 | 7.90 | 118.89 | 7.87 | 118.47 | 7.87 | 118.32 | 7.84 | 118.56 | 7.87 |
| 78 | **GLU** |  |  | 118.78 | 7.53 | 118.76 | 7.51 | 119.52 | 7.51 | 119.48 | 7.74 |
| 79 | **GLU** | 118.55 | 8.15 | 119.10 | 7.87 | 118.91 | 7.87 | 118.37 | 7.82 |  |  |
| 80 | **CYS** | 118.72 | 8.41 | 118.51 | 8.48 | 119.41 | 8.33 | 121.08 | 8.42 |  |  |
| 81 | **LEU** | 119.73 | 7.59 | 119.56 | 7.54 | 119.81 | 7.24 | 120.04 | 7.09 |  |  |
| 82 | **HIS** |  |  | 119.24 | 8.08 | 117.14 | 7.85 | 116.28 | 7.84 |  |  |
| 83 | **ARG** |  |  |  |  | 119.03 | 7.77 | 118.70 | 7.69 |  |  |
| 84 | **ILE** |  |  |  |  |  |  | 130.28 | 8.71 |  |  |
| 85 | **ARG** |  |  |  |  |  |  |  |  |  |  |
| 86 | **LYS** |  |  |  |  |  |  |  |  |  |  |
| 87 | **GLU** |  |  |  |  |  |  |  |  |  |  |
| 88 | **ARG** |  |  |  |  |  |  |  |  |  |  |
| 89 | **TRP** | 118.74 | 7.97 | 119.14 | 8.01 | 119.07 | 8.02 | 118.89 | 8.06 |  |  |
| 89 | TRP | 129.54 | 10.18 | 129.60 | 10.17 | 129.64 | 10.34 | 130.39 | 10.68 |  |  |
| 90 | **GLY** | 109.98 | 7.95 | 109.77 | 7.94 | 106.5 | 7.74 | 102.78 | 7.66 |  |  |
| 91 | **PRO** |  |  |  |  |  |  |  |  |  |  |
| 92 | **ARG** |  |  |  |  |  |  | 120.87 | 9.50 |  |  |
| 93 | **THR** |  |  |  |  | 113.87 | 8.22 | 116.93 | 6.42 |  |  |
| 94 | **LEU** |  |  |  |  | 115.48 | 9.34 | 114.98 | 8.35 |  |  |
| 95 | **ASP** | 125.95 | 8.78 | 124.53 | 8.44 | 126.68 | 8.60 | 124.92 | 8.19 |  |  |
| 96 | **VAL** | 117.97 | 10.26 | 129.93 | 10.41 | 129.16 | 10.33 | 129.94 | 10.40 |  |  |
| 97 | **ASP** | 124.49 | 9.23 | 126.66 | 9.32 | 126.83 | 9.39 | 127.78 | 9.39 |  |  |
| 98 | **ILE** | 122.86 | 9.41 | 125.87 | 10.94 | 126.18 | 10.96 | 126.42 | 10.81 |  |  |
| 99 | **LEU** | 127.53 | 9.00 | 126.84 | 9.11 | 126.95 | 9.10 | 127.26 | 9.00 | 127.68 | 9.02 |
| 100 | **LEU** | 113.09 | 7.46 | 113.47 | 7.58 | 113.34 | 7.56 | 113.01 | 7.50 | 113.10 | 7.44 |
| 101 | **TYR** | 125.29 | 9.41 | 125.30 | 9.44 | 125.33 | 9.43 | 125.23 | 9.42 | 125.34 | 9.40 |
| 102 | **GLY** | 114.37 | 8.06 | 114.23 | 8.14 | 114.15 | 8.13 | 114.22 | 8.13 | 114.35 | 8.03 |
| 103 | **GLU** | 127.06 | 9.15 | 128.35 | 9.28 | 128.34 | 9.28 | 128.28 | 9.26 | 127.09 | 9.16 |
| 104 | **GLU** | 118.58 | 8.10 | 118.80 | 8.17 | 118.77 | 8.18 | 118.62 | 8.15 | 118.51 | 8.09 |
| 105 | **MET** | 119.02 | 8.37 | 118.55 | 8.28 | 118.49 | 8.28 | 118.41 | 8.27 | 119.03 | 8.36 |
| 106 | **ILE** | 126.12 | 9.39 | 126.57 | 9.71 | 126.50 | 9.70 | 126.55 | 9.69 | 126.08 | 9.40 |
| 107 | **ASP** | 128.72 | 8.82 | 128.73 | 8.83 | 128.76 | 8.85 | 128.85 | 8.86 | 128.78 | 8.84 |
| 108 | **LEU** | 124.93 | 8.52 | 124.82 | 8.69 | 124.82 | 8.69 | 124.64 | 8.66 | 124.76 | 8.51 |
| 109 | **PRO** |  |  |  |  |  |  |  |  |  |  |
| 110 | **LYS** | 122.01 | 8.48 | 121.63 | 7.96 | 121.48 | 8.04 | 121.51 | 8.02 | 121.88 | 8.47 |
| 111 | **LEU** | 119.69 | 7.37 | 119.96 | 7.43 | 119.97 | 7.43 | 120.12 | 7.44 | 119.58 | 7.35 |
| 112 | **SER** | 120.95 | 8.26 | 124.80 | 9.25 | 124.84 | 9.29 | 124.53 | 9.20 | 121.15 | 8.25 |
| 113 | **VAL** | 123.86 | 8.92 | 124.69 | 8.82 | 124.87 | 8.83 | 125.08 | 8.85 | 123.87 | 8.93 |
| 114 | **PRO** |  |  |  |  |  |  |  |  |  |  |
| 115 | **HIS** |  |  | 127.03 | 8.67 | 126.79 | 8.60 | 126.41 | 8.47 |  |  |
| 116 | **PRO** |  |  |  |  |  |  |  |  |  |  |
| 117 | **ARG** |  |  | 118.71 | 9.62 | 118.36 | 9.64 | 118.7 | 9.73 |  |  |
| 118 | **MET** | 119.98 | 7.79 | 119.77 | 7.50 | 119.48 | 7.55 | 119.76 | 7.55 | 119.71 | 7.72 |
| 119 | **ASN** | 112.64 | 8.12 | 113.81 | 8.10 | 114.22 | 8.08 | 113.71 | 8.03 | 112.95 | 8.10 |
| 120 | **GLU** | 115.57 | 7.54 | 114.64 | 7.33 | 114.44 | 7.34 | 114.41 | 7.31 | 115.16 | 7.48 |
| 121 | **ARG** | 120.20 | 7.34 | 120.13 | 7.17 | 121.51 | 7.19 | 121.68 | 7.08 | 120.65 | 7.31 |
| 122 | **ALA** | 131.66 | 9.55 | 132.18 | 9.78 | 153.79 | 9.38 | 132.69 | 9.36 | 132.37 | 9.33 |
| 123 | **PHE** | 107.83 | 6.62 | 108.60 | 6.68 | 112.23 | 8.49 | 111.88 | 8.20 |  |  |
| 124 | **VAL** | 118.03 | 6.35 | 118.82 | 6.37 | 118.72 | 6.36 | 118.80 | 6.30 | 118.09 | 6.28 |
| 125 | **LEU** | 115.01 | 7.94 | 115.01 | 7.94 | 115.41 | 8.02 | 115.35 | 7.88 | 114.93 | 7.84 |
| 126 | **LEU** | 115.30 | 8.14 | 115.32 | 8.21 | 115.69 | 8.43 | 115.69 | 8.38 | 115.50 | 8.26 |
| 127 | **PRO** |  |  |  |  |  |  |  |  |  |  |
| 128 | **LEU** | 117.70 | 7.88 | 117.38 | 7.91 | 117.53 | 8.14 | 117.50 | 8.11 | 117.75 | 7.99 |
| 129 | **ASN** | 118.52 | 8.40 | 119.01 | 8.51 | 118.97 | 8.50 | 118.89 | 8.46 | 118.70 | 8.43 |
| 130 | **ASP** | 115.31 | 7.23 | 115.55 | 7.26 | 115.32 | 7.22 | 115.14 | 7.19 | 115.03 | 7.18 |
| 131 | **ILE** | 106.55 | 6.99 | 106.53 | 6.99 |  |  | 106.51 | 7.00 | 106.51 | 7.00 |
| 132 | **ALA** | 128.34 | 8.55 | 128.57 | 8.56 | 128.43 | 8.55 | 128.20 | 8.50 | 127.96 | 8.50 |
| 133 | **ALA** | 120.59 | 7.38 | 120.59 | 7.42 | 120.63 | 7.44 | 120.59 | 7.41 | 120.69 | 7.37 |
| 134 | **ASN** | 107.83 | 8.13 | 107.86 | 8.14 | 86.93 | 8.15 | 107.81 | 8.14 | 107.89 | 8.14 |
| 135 | **VAL** | 123.63 | 7.56 | 123.52 | 7.53 | 123.58 | 7.53 | 123.58 | 7.53 | 123.67 | 7.56 |
| 136 | **VAL** | 125.95 | 8.15 | 125.86 | 8.14 | 125.90 | 8.15 | 125.90 | 8.14 | 125.99 | 8.16 |
| 137 | **GLU** | 134.40 | 8.92 | 134.44 | 8.89 | 155.47 | 8.88 | 134.33 | 8.87 | 134.58 | 8.91 |
| 138 | **PRO** |  |  |  |  |  |  |  |  |  |  |
| 139 | **ARG** | 121.16 | 9.46 | 120.75 | 9.48 | 120.83 | 9.49 | 120.94 | 9.48 | 121.08 | 9.46 |
| 140 | **SER** | 116.43 | 9.83 | 116.64 | 9.88 | 116.72 | 9.92 | 116.52 | 9.83 | 116.42 | 9.85 |
| 141 | **LYS** | 116.90 | 8.36 | 116.79 | 8.41 | 116.82 | 8.41 | 116.67 | 8.38 | 116.79 | 8.33 |
| 142 | **LEU** | 121.59 | 8.05 | 121.68 | 8.04 | 121.68 | 8.06 | 121.64 | 8.04 | 121.53 | 8.04 |
| 143 | **LYS** | 118.31 | 8.57 | 118.21 | 8.56 | 118.25 | 8.56 | 118.21 | 8.55 | 118.34 | 8.58 |
| 144 | **VAL** | 123.47 | 8.38 | 123.58 | 8.41 | 123.64 | 8.41 | 123.54 | 8.39 | 123.48 | 8.35 |
| 145 | **LYS** | 115.01 | 8.86 | 114.99 | 8.88 | 115.00 | 8.91 | 114.87 | 8.88 | 115.07 | 8.90 |
| 146 | **ASP** | 120.18 | 7.83 | 120.27 | 7.84 | 120.31 | 7.84 | 120.22 | 7.83 | 120.32 | 7.84 |
| 147 | **LEU** | 121.02 | 7.82 | 121.50 | 7.79 | 121.54 | 7.78 | 121.48 | 7.77 | 121.06 | 7.79 |
| 148 | **VAL** | 117.80 | 7.17 | 117.83 | 7.20 | 117.95 | 7.23 | 117.90 | 7.19 | 117.85 | 7.17 |
| 149 | **PHE** | 122.24 | 7.32 | 122.22 | 7.36 | 122.40 | 7.49 | 122.47 | 7.48 | 122.24 | 7.44 |
| 150 | **VAL** | 120.75 | 8.48 | 120.87 | 8.50 | 121.05 | 8.49 | 121.17 | 8.47 | 121.04 | 8.48 |
| 151 | **ASP** | 125.78 | 8.11 | 125.60 | 8.19 | 125.53 | 8.19 | 125.56 | 8.18 | 125.68 | 8.13 |
| 152 | **ASP** | 124.96 | 8.84 | 124.96 | 8.86 | 125.27 | 8.85 | 125.16 | 8.82 | 125.32 | 8.83 |
| 153 | **SER** | 115.30 | 8.96 | 115.23 | 8.93 | 116.27 | 9.15 | 116.19 | 9.12 | 116.33 | 9.17 |
| 154 | **VAL** | 123.93 | 7.62 | 123.97 | 7.63 | 124.95 | 7.57 | 124.97 | 7.53 | 124.83 | 7.60 |
| 155 | **LYS** | 125.20 | 8.84 | 124.98 | 8.82 | 125.35 | 8.73 | 125.29 | 8.70 | 125.29 | 8.76 |
| 156 | **ARG** | 124.89 | 9.10 | 124.94 | 9.12 | 126.35 | 9.26 | 126.03 | 9.23 | 126.08 | 9.23 |
| 157 | **TYR** | 129.70 | 9.04 | 129.70 | 9.03 | 128.97 | 8.94 | 128.81 | 8.94 | 129.02 | 8.97 |
| 158 | **LYS** | 123.86 | 7.68 | 124.01 | 7.65 | 124.09 | 7.73 | 123.88 | 7.73 | 123.54 | 7.70 |
